# Influenza virus infection activates TAK1 to suppress RIPK3-independent apoptosis and RIPK1-dependent necroptosis

**DOI:** 10.1186/s12964-024-01727-2

**Published:** 2024-07-23

**Authors:** Yuling Sun, Lei Ji, Wei Liu, Jing Sun, Penggang Liu, Xiaoquan Wang, Xiufan Liu, Xiulong Xu

**Affiliations:** 1 College of Veterinary Medicine, Institute of Comparative Medicine, Yangzhou University, Yangzhou, Jiangsu Province 225009 P. R. China; 2 Animal Infectious Disease Laboratory, College of Veterinary Medicine, Yangzhou University, Yangzhou, 225009 China; 3 Jiangsu Co-innovation Center for Prevention and Control of Important Animal Infectious Diseases and Zoonosis, Yangzhou University, Yangzhou, Jiangsu Province 225009 China

**Keywords:** Influenza A virus, Apoptosis, Necroptosis, TAK1, RIPK1

## Abstract

Many DNA viruses develop various strategies to inhibit cell death to facilitate their replication. However, whether influenza A virus (IAV), a fast-replicating RNA virus, attenuates cell death remains unknown. Here, we report that IAV infection induces TAK1 phosphorylation in a murine alveolar epithelial cell line (LET1) and a murine fibroblastoma cell line (L929). The TAK1-specific inhibitor 5Z-7-Oxzeneonal (5Z) and TAK1 knockout significantly enhance IAV-induced apoptosis, as evidenced by increased PARP, caspase-8, and caspase-3 cleavage. TAK1 inhibition also increases necroptosis as evidenced by increased RIPK1^S166^, RIPK3^T231/S232^, and MLKL^S345^ phosphorylation. Mechanistically, TAK1 activates IKK, which phosphorylates RIPK1^S25^ and inhibits its activation. TAK1 also activates p38 and its downstream kinase MK2, which phosphorylates RIPK1^S321^ but does not affect RIPK1 activation. Further investigation revealed that the RIPK1 inhibitor Nec-1 and RIPK1 knockout abrogate IAV-induced apoptosis and necroptosis; re-expression of wild-type but not kinase-dead (KD)-RIPK1 restores IAV-induced cell death. ZBP1 knockout abrogates IAV-induced cell death, whereas RIPK3 knockout inhibits IAV-induced necroptosis but not apoptosis. 5Z treatment enhances IAV-induced cell death and slightly reduces the inflammatory response in the lungs of H1N1 virus-infected mice and prolongs the survival of IAV-infected mice. Our study provides evidence that IAV activates TAK1 to suppress RIPK1-dependent apoptosis and necroptosis, and that RIPK3 is required for IAV-induced necroptosis but not apoptosis in epithelial cells.

**Supplementary Information:**

The online version contains supplementary material available at 10.1186/s12964-024-01727-2.

## Introduction

Influenza A virus (IAV) is a highly contagious pathogen that belongs to the *Orthomyxoviridae* family. H1N1 and H3N2 viruses are the prevalent subtypes that cause seasonal epidemics and hundreds of thousands of deaths annually [1]. The 2009 influenza pandemic claimed 150 to 600 thousand lives [2]. H5N1 and its derivative subtypes (H5NX) are highly pathogenic strains of IAV of avian origin that can cause sporadic fatal infections in humans [3, 4]. IAV replicates rapidly in bronchial and alveolar epithelial cells and induces multiple forms of cell death including apoptosis, necroptosis, and pyroptosis [5–7]. The PB1-F2 protein of IAV can target the mitochondrial proteins, adenine nucleotide translocase 3 (ANT3) and voltage-dependent anion channel 1 (VDAC1), to release cytochrome C and induce intrinsic apoptosis [8, 9]. In addition, IAV up-regulates Fas ligand and TNF-related apoptosis-inducing ligand (TRAIL) expression and induces extrinsic apoptosis [8].

ZBP1 (Z-form RNA binding protein 1 (ZBP1)/DNA activator of interferons (DAI)) contains two Zα domains for detecting the Z-from nucleic acid and a RHIM (RIP homotypic interaction motif) domain that interacts with other proteins with the RHIM structure [10]. ZBP1 has been implicated in playing a central role in IAV-induced cell death [11–13]. Upon binding to the viral genomic RNA of IAV, ZBP1 engages with RIPK3 to activate it (MEFs) [14, 15]. RIPK3 then phosphorylates mixed lineage kinase domain-like protein (MLKL) at S345 and induces its oligomerization and translocation into the cell membrane where it forms pores to induce necroptosis [14, 15]. RIPK3 also interacts with RIPK1 to form the RIPK3-RIPK1-FADD-caspase-8 complex to activate caspase-8 and induce apoptosis in IAV-infected MEFs [14, 15]. In this complex, RIPK1 functions as a scaffolding protein, its kinase activity is dispensable for IAV-induced apoptosis in MEFs [14, 15]. ZBP1 is constitutively expressed in MEFs at high levels [14, 15]. In contrast, ZBP1 is not expressed in epithelial cells but can be induced by interferons. Whether IAV induces apoptosis and necroptosis in airway epithelial cells via the same pathways as in MEFs, which are not a type of host cells for IAV, remains unknown.

TGF-β-activated kinase 1 (TAK1) is a member of the MAP3K family that can be activated by a variety of intra- and extracellular stimuli such as TNF-α, TRAIL, and LPS [16, 17]. Through its interaction with ubiquitinated RIPK1, TAK1 is assembled into the TNF-α receptor-associated complex I where it binds to TAB1 and TAB2/TAB3 and becomes ubiquitinated and phosphorylated at multiple sites of its kinase domain [18, 19]. In addition, TAK1 can be directly activated by viral proteins such as the gp41 glycoprotein of HIV-1 or indirectly by TLRs [20–22]. TAK1 plays a central role in maintaining cell viability and tissue homeostasis in a variety of organs [17]. Activated TAK1 phosphorylates IKKγ in the IKK complex to activate NF-κB and exert its prosurvival role [16–19]. Recent studies have shown that TAK1 may directly phosphorylate RIPK1 or indirectly phosphorylate RIPK1 through p38-activated MK2 at serine 321 (RIPK1^S321^) (mouse) or RIPK1^S320^ (human) to inhibit its activity and inhibit TNF-α-induced apoptosis and necroptosis [23–27]. TAK1 also activates IKK to phosphorylate RIPK1^S25^ and inhibit RIPK1 activity [28, 29]. Whether TAK1 regulates IAV-induced cell death remains unexplored.

Programmed cell death is thought to be an integral part of host cell defense against invading intracellular microbes [8, 30]. Virus replication is terminated when host cells undergo programmed cell death, particularly apoptosis. Several DNA viruses such as murine cytomegalovirus, vaccinia, and cowpox virus express a caspase inhibitor and a RHIM-containing viral protein to subvert apoptosis and necroptosis, respectively [33–33]. Whether IAV-induced apoptosis and necroptosis are repressed or delayed is incompletely understood. Our present study aims at understanding the mechanisms of IAV-induced suppression of cell death, whether IAV induces cell death by different pathways between MEFs and non-MEFs. Here we report that IAV infection rapidly activated TAK1 to inhibit RIPK1 activity and cell death by IKK but not by p38-activated MK2. RIPK1 and ZBP1 are required for IAV-induced necroptosis and apoptosis, whereas RIPK3 is required for IAV-induced necroptosis but not apoptosis. Our study suggests that TAK1 functions as a master regulator to prevent premature cell death and that RIPK1 is indispensable in IAV-induced apoptosis and necroptosis.

## Results

### IAV induces apoptosis and necroptosis

**Fig. 1:**
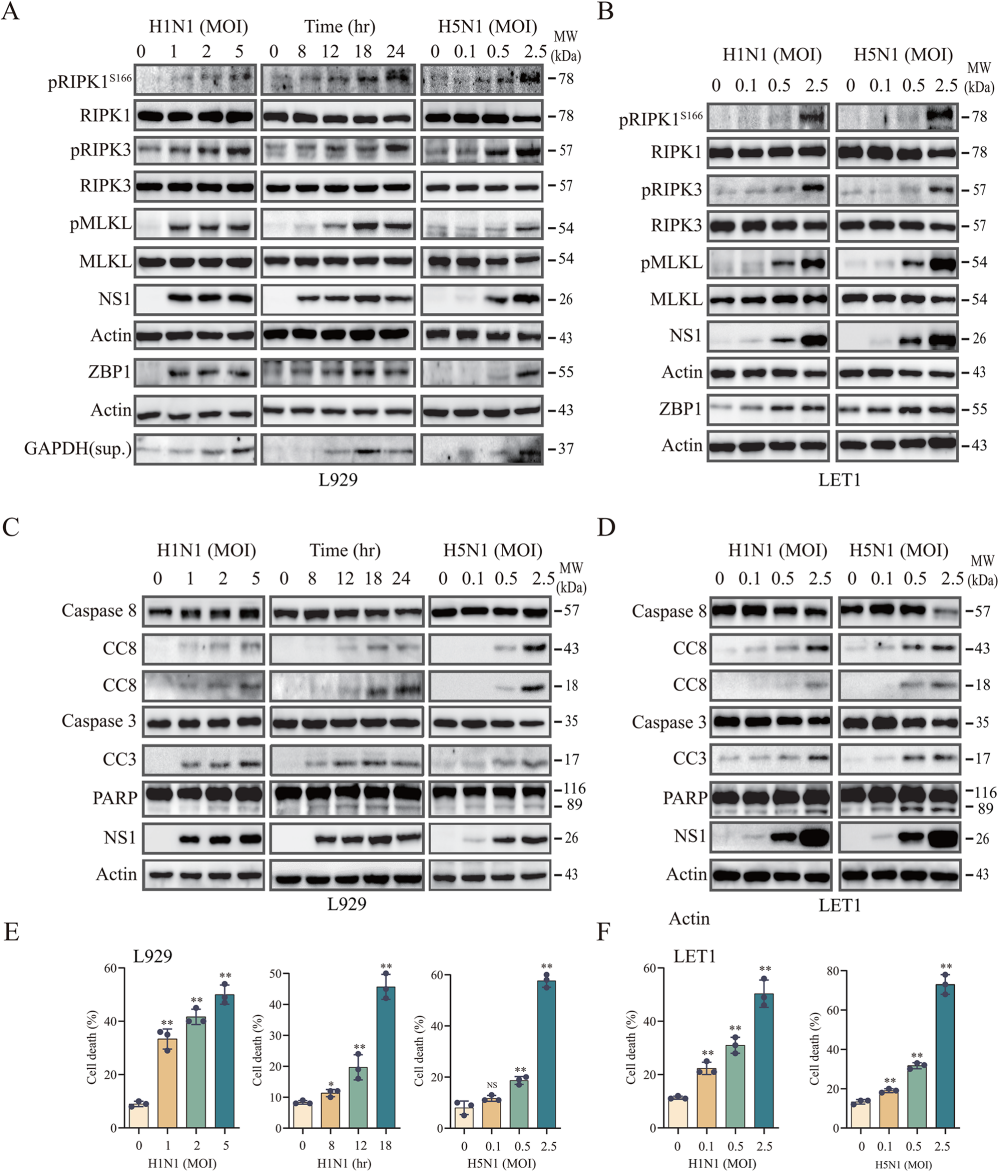
IAV induces cell death. **(A-D)** L929 and LET1 cells were infected with the indicated MOI of H1N1 or H5N1 (SY strain) for 18 h or with 2 MOI of H1N1 virus for the indicated lengths of time. Cell lysates were analyzed for the activation of the necroptotic pathway (**A** & **B**) by detecting RIPK1^S166^, RIPK3, and MLKL phosphorylation, their corresponding total proteins, NS1, ZBP1, and β-actin as a loading control. GAPDH in the conditioned media of L929 cells was also probed. (**C** & **D**) Cell lysates were analyzed for the levels of caspase-8, cleaved caspase-8 (CC8) (43- and 18-kDa), caspase-3, cleaved caspase-3 (CC3) (17-kDa), PARP, NS1, and β-actin. L929 (**E**) and LET1 (**F**) cells seeded in a 96-well plate were infected with the indicated MOI of H1N1 or H5N1 (SY strain) for 18 h or infected with 2 MOI of H1N1 virus and then incubated for the indicated lengths of time. Cells were labelled with PI (2 µg/ml) and then read in a plate reader. Data represents the mean ± SD of three independent experiments. Unpaired Student’s *t* test was used to determine the significant difference between un-infected and infected cells. * *p* < 0.05, ** *p* < 0.01

Prior studies have shown that the murine-adapted H1N1 virus (PR8) readily induces necroptosis in MEFs, as evidenced by increased MLKL phosphorylation at S345 [14, 15, 34]. We hypothesized that IAV infection may induce apoptosis and necroptosis in airway epithelial cells by the pathways that are different in MEFs, a type of non-host cells for IAV. LET1 cells, a murine noncancerous alveolar epithelial cell line, were chosen for this purpose. In addition, L929 cells, a murine fibroblastoma cell line that has been widely used for investigating necroptosis under various settings including virus infections, were also used as a control [35]. We first tested the feasibility of these two cell lines in inducing necroptosis by analyzing RIPK1^S166^, RIPK3^T231/S232^, and MLKL^S345^ phosphorylation. The antibodies used here were carefully chosen, they all have a proven specificity according to a recent publication [36]. The H1N1 (PR8) and H5N1 virus (SY) induced RIPK1, RIPK3, and MLKL phosphorylation in L929 and LET1 cells in a dose- and time-dependent manner (Fig. 1A & B). ZBP1 was expressed at a low level in uninfected LET1 cells but not in L929 cells (Fig. 1A & B). However, ZBP1 expression was induced in both cell lines infected with H5N1 or H1N1 virus (Fig. 1A & B). Necroptotic L929 cells release glyceraldehyde 3-phosphate dehydrogenase (GAPDH) in their conditioned media [37]. Detection of GAPDH in the conditioned media of IAV-infected L929 cells can serve as a marker of necroptosis [37]. GAPDH levels were elevated in a time- and dose-dependent manner in the conditioned media of H1N1 virus-infected L929 cells, which further indicates necroptosis (Fig. 1A).

Caspase-8 functions as an initiator caspase to activate the effector caspase-3, which then cleaves and inactivates poly (ADP-ribose) polymerase-1 (PARP1) to induce apoptosis [38]. To detect IAV-induced apoptosis, we assessed the cleavage of these two caspases and PARP in IAV-infected LET1 and L929 cells. Both H1N1 and H5N1 virus infections induced caspase-8, caspase-3, and PARP cleavage in a dose- and time-dependent manner in L929 and LET1 cells (Fig. 1C & D). Caspase-8 was cleaved twice to generate an 18-kDa protein and a 43-kDa protein, whereas caspase-3 was cleaved to create a dominant 17-kDa protein in L929 cells. A minor 19-kDa caspase-3 fragment was occasionally visible in some blots. PARP was cleaved to generate an 89-kDa protein. Propidium iodide (PI) is a small fluorescent molecule that binds to DNA but cannot passively traverse into cells with an intact plasma membrane. PI uptake versus exclusion can be utilized to discriminate dead cells from live cells. H1N1 and H5N1 virus infection increased the fluorescent intensity of PI-stained dead L929 and LET1 cells (Fig. 1E & F).

L929 cells are a murine fibroblastoma cell line that produces a large quantity of IFNs and inflammatory cytokines including TNF-α and has been widely used for investigating TNF-α-induced necroptosis [39]. IAV infection readily increases the production of TNF-α. To rule out the possibility that IAV-induced cell death was indirectly mediated through the TNF receptor, we first determined if an anti-TNF-α neutralizing antibody could attenuate IAV-induced cell death in L929 cells. IAV was able to fully induce RIPK1, RIPK3, and MLKL phosphorylation and caspase-3, caspase-8, and PARP cleavage in the presence of a neutralizing anti-TNF-α antibody in L929 cells (Fig. S1A & B). To ascertain the efficiency of the anti-TNF-α antibody in blocking TNF-α-induced cell death, TSZ (TNF-α, Smac mimetic, and Z-VAD-FMK), a cocktail that inhibits caspase activity and induces necroptosis, was included as a positive control. Indeed, the anti-TNF-α antibody alleviated TSZ-induced RIPK1, RIPK3, and MLKL phosphorylation in L929 cells (Fig. S1A & B). TNFR knockdown in LET1 cells did not affect the ability of H1N1 virus to induce MLKL phosphorylation and caspase-3 cleavage but drastically reduced TSZ-induced MLKL phosphorylation and caspase-3 cleavage (Fig. S1C), further suggesting that IAV-induced cell death is independent of the TNF-α receptor pathway.

### IAV increases TAK1 and RIPK1^S321^ phosphorylation

**Fig. 2:**
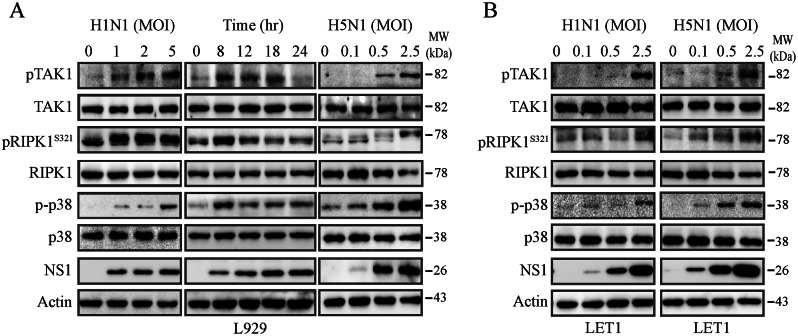
IAV infection activates TAK1. L929 (**A**) and LET1 (**B**) cells were infected with various MOI of H1N1 (PR8 strain) or H5N1 (SY strain) viruses for 18 h or with H1N1 virus (2 MOI) for the indicated lengths of time. Cell lysates were analyzed for the levels of TAK1, RIPK1^S321^, and p38 phosphorylation and their corresponding total proteins by Western blot. The levels of NS1 and β-actin as a loading control were also detected

TAK1 activation leads to phosphorylation of several downstream signaling molecules including RIPK1^S321^, NF-κB, JNK, p38, and AMPK and plays important roles in innate immunity and cell death [19, 40]. TAK1 can activate p38 and MK2 to phosphorylate RIPK1 S321 (mouse) and inhibit its activation [23]. We first investigated if IAV was able to activate TAK1 and phosphorylate RIPK1 S321 in these two cell lines. IAV infection dose-dependently increased TAK1 and p38 phosphorylation in L929 (Fig. 2A) and LET1 cells (Fig. 2B). Intriguingly, RIPK1 phosphorylation at S321 was readily visible in uninfected L929 cells but was remarkably increased following IAV infection. However, S321-phosphorylated RIPK1 in IAV-infected L929 cells had a slower electrophoresis mobility than that in uninfected cells (Fig. 2A). This is likely due to additional phosphorylation such as at S166. IAV infection increased TAK1 and p38 phosphorylation in L929 cells as early as 8 h post infection (hpi) (Fig. 2A). H1N1 virus infection significantly increased RIPK1^S321^ phosphorylation, leading to the upper shift of the RIPK1 band in L929 cells (Fig. 2A), a phenomenon also observed by others [23].

### TAK1 inhibition suppresses RIPK1^S321^ phosphorylation but activates RIPK1 to potentiate IAV-induced apoptosis and necroptosis

**Fig. 3:**
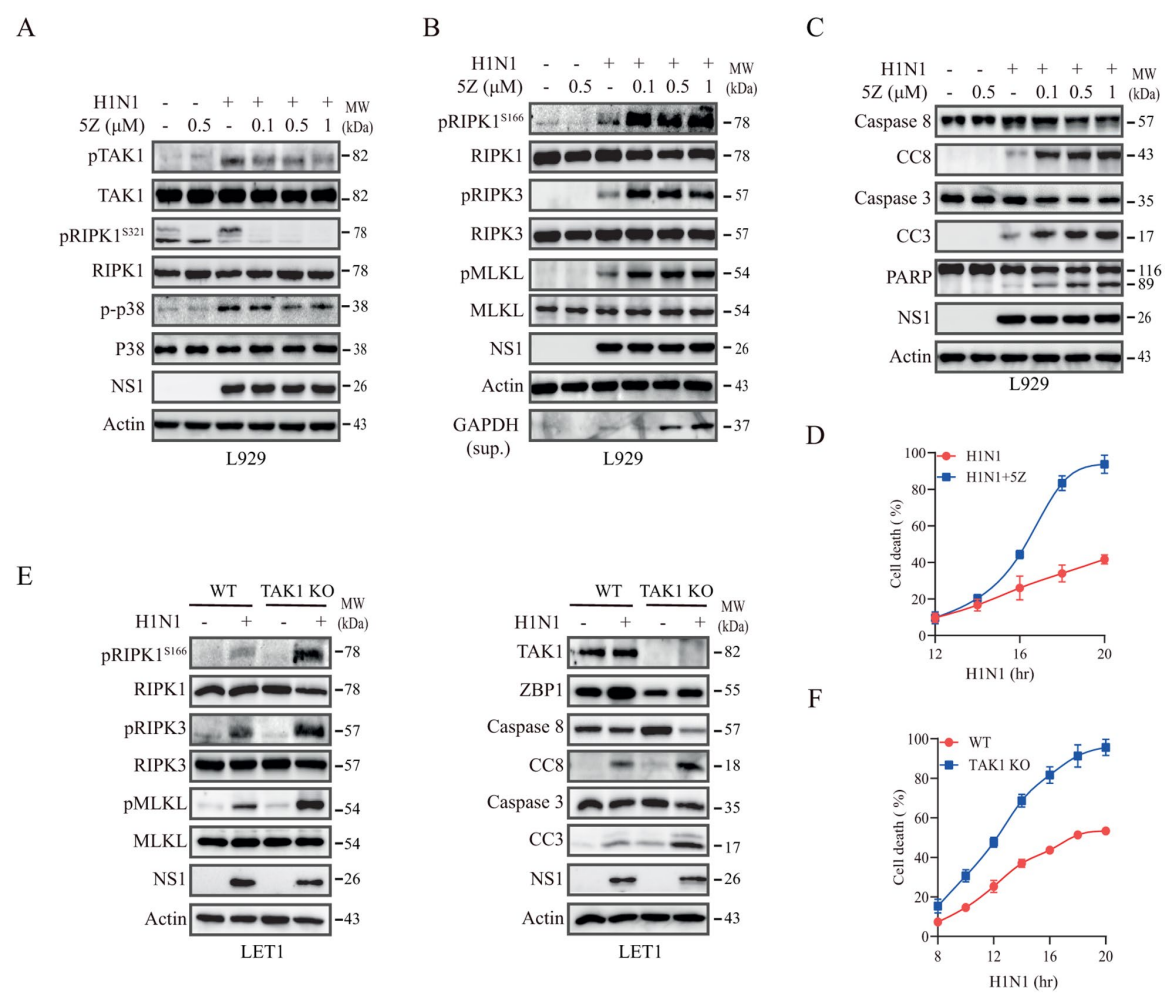
TAK1 inhibition enhances IAV-induced RIPK1 activation and potentiates apoptosis and necroptosis. (**A-C**) L929 cells were infected with H1N1 virus (2 MOI). After incubation for 12 h, 5Z was added at the indicated concentrations and then incubated for another 6 h. Cell lysates were analyzed for the levels of TAK1, RIPK1^S321^, and p38 phosphorylation and their corresponding total protein (**A**) or analyzed for the activation of necroptosis (**B**) or apoptosis (**C**) by Western blot. NS1 as a virus infection control and β-actin as a loading control were detected. GAPDH in the conditioned media indicating necroptosis was also probed. (**D**) L929 cells seeded in a 96-well plate were infected with 2 MOI of H1N1. After incubation for 12 h, 5Z (0.5 µM) was added. Cells labelled with PI (2 µg/ml) were read at the indicated time in a plate reader. (**E**) TAK1 knockout enhances necroptosis and apoptosis. Control and TAK1 knockout LET1 cells were infected with H1N1 viruses (2 MOI) and then incubated for 18 h. Cell lysates were analyzed for the levels of necroptosis-related protein phosphorylation (RIPK1^S166^, RIPK3, and MLKL), actin, TAK1, ZBP1, and apoptosis-related proteins by Western blot. (**F**) Control and TAK1 knockout LET1 cells seeded in a 96-well plate were infected with 2 MOI of H1N1. Cells labelled with PI (2 µg/ml) were read at the indicated time in a plate reader. Data represents the mean ± SD of the triplicate from one of three independent experiments with similar results (**D** & **F**)

We then determined if TAK1 activation was responsible for RIPK1^S321^ phosphorylation. The TAK1 inhibitor 5Z used at very low concentrations (0.1, 0.5, and 1 µM) inhibited H1N1 virus-induced RIPK1^S321^ phosphorylation in L929 cells in a dose-dependent manner (Fig. 3A). 5Z less effectively inhibited p38 phosphorylation than RIPK1^S321^ phosphorylation in L929 cells (Fig. 3A). 5Z increased the levels of RIPK1^S166^, RIPK3, and MLKL phosphorylation and the release of GAPDH into the conditioned media of H1N1-infected L929 (Fig. 3B). 5Z increased PARP, caspase-8, and caspase-3 cleavage effectively in H1N1 virus-infected L929 cells (Fig. 3C). PI staining revealed that 5Z increased H1N1 virus-induced cell death in L929 cells in a time-dependent manner (Fig. 3D). Consistent with these observations, TAK1 knockout significantly increased H1N1 virus-induced RIPK1^S166^, RIPK3, and MLKL phosphorylation as well as caspase-8 and caspase-3 cleavage, albeit ZBP1 expression was reduced in TAK1-deficienct LET1 cells (Fig. 3E). TAK1 knockout also time-dependently increased H1N1 virus-induced cell death in LET1 cells (Fig. 3F).

### TAK1 suppresses RIPK1 activation and cell death by IKK but not by p38-activated MK2

**Fig. 4:**
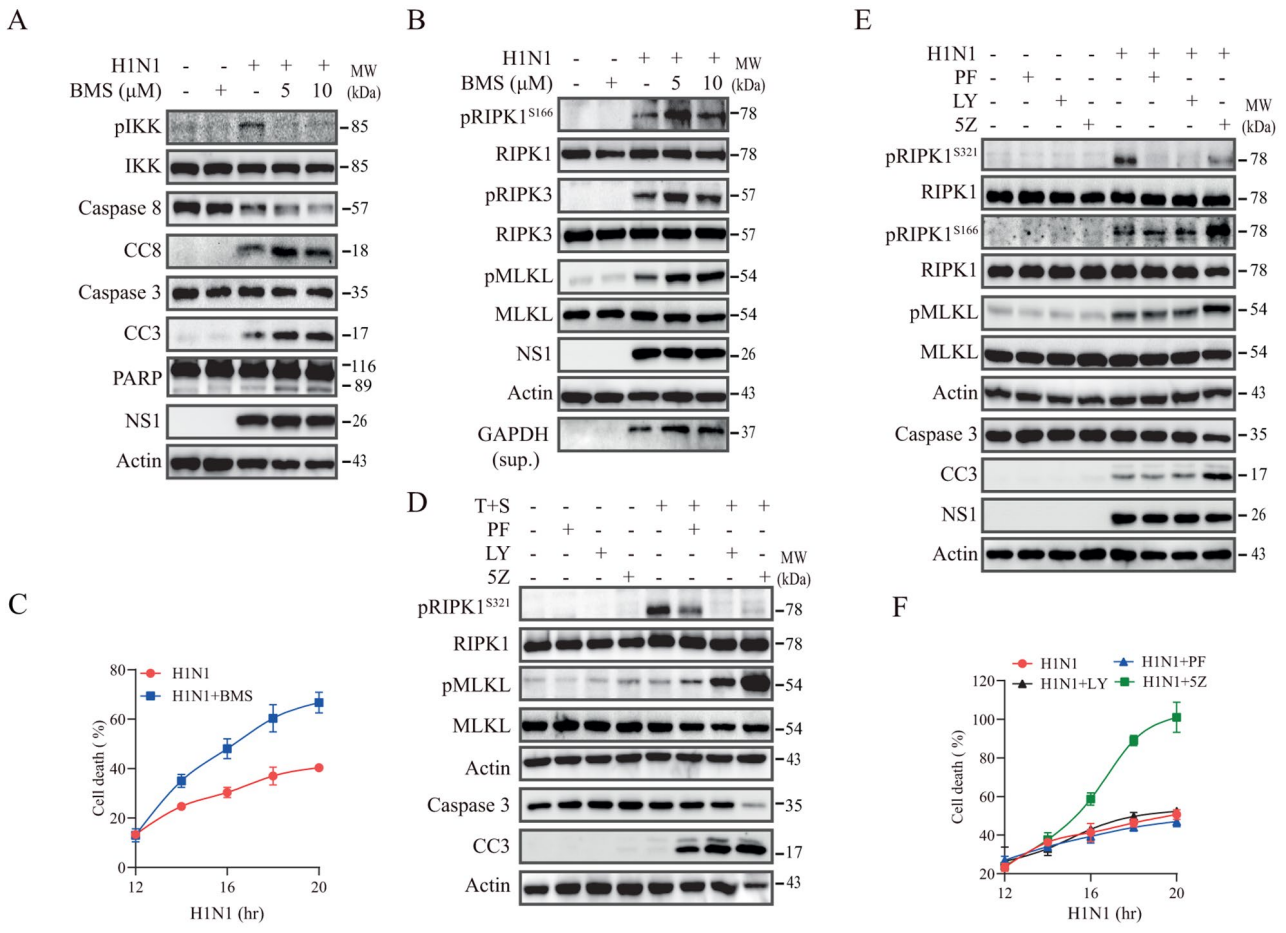
TAK1 suppresses RIPK1 activation by IKK but not by p38/MK2. (**A** & **B**) L929 cells were infected with H1N1 viruses (2 MOI). After incubation for 12 h, BMS was added at the indicated concentrations and then incubated for another 12 h. Cell lysates were analyzed for the levels of IKK phosphorylation, caspase-3, and -8, CC3, CC8, PARP, NS1, and β-actin by Western blot (**A**). (**B**) Alternatively, cell lysates were analyzed for the levels of RIPK1, RIPK3, and MLKL phosphorylation and their corresponding total protein by Western blot. GAPDH in the conditioned media was also probed. (**C**) L929 cells seeded in a 96-well plate were infected with the indicated MOI of H1N1. After incubation for 12 h, BMS (10 µM) was added. Cells labelled with PI (2 µg/ml) were read at the indicated time in a plate reader. (**D**) L929 cells were treated with PF (1 µM), LY (1 µM) or 5Z (0.5 µM). After incubation for 1 h, TNF-α (20 ng/ml) and Smac (100 nM) were added and then incubated for another 4 h. Cell lysates were analyzed for the levels of RIPK1^S321^ and MLKL phosphorylation, caspase-3 cleavage and actin by Western blot. (**E**) L929 cells were infected with H1N1 virus (2 MOI). After incubation for 12 h, PF (1 µM), LY (1 µM) or 5Z (0.5 µM) was added and then incubated for another 6 h. Cell lysates were analyzed for the levels of RIPK1^S321^, RIPK1^S166^ and MLKL phosphorylation, caspase-3 cleavage, NS1, and actin by Western blot. (**F**) L929 cells seeded in a 96-well plate were infected with the indicated MOI of H1N1. After incubation for 12 h, PF (1 µM), LY (1 µM) or 5Z (0.5 µM) was added and then incubated in the absence or presence of LY, PF or 5Z. Cells labelled with PI (2 µg/ml) were read at the indicated time in a plate reader. Data represents the mean ± SD of the triplicate from one of three independent experiments with similar results (**C** & **F**)

TAK1 suppresses RIPK1 activation by activating IKK to phosphorylate RIPK1 at S25 or by activating p38 and its downstream MK2 kinase to phosphorylate RIPK1 at S321 [35]. Here we tested if inhibition of RIPK1 activation in IAV-infected cells was mediated by IKK or by p38-activated MK2. BMS345541 (BMS), an IKK-specific inhibitor, inhibited IAV-induced IKK phosphorylation but increased caspase-8, caspase-3, and PARP cleavage in L929 cells (Fig. 4A). BMS also increased IAV-induced RIPK1^S166^, RIPK3, and MLKL phosphorylation in L929 cells (Fig. 4B). Of note, RIPK1^S25^ phosphorylation was not analyzed due to the lack of a commercially available antibody. Again, BMS sensitized L929 to H1N1 virus-induced cell death (Fig. 4C). We next determine if TAK1 activation suppressed RIPK1 activation also by TAK1-activated p38 and MK2. LY2228820 (LY), a p38-specific inhibitor, and PF-3644022 (PF), a MK2-specific inhibitor, inhibited TNF-α/Smac-induced and IAV-induced RIPK1^S321^ phosphorylation (Fig. 4D & E) and enhanced TNF-α-induced caspase cleavage as well as MLKL phosphorylation (Fig. 4D). However, these two inhibitors did not increase H1N1 virus-induced MLKL phosphorylation and caspase-3 cleavage (Fig. 4E). Consistently, LY and PF did not enhance H1N1 virus-induced cell death in L929 cells (Fig. 4F). These observations collectively suggest that IAV activates TAK1 to suppress RIPK1 activation not by p38/MK2-mediated RIPK1^S321^ phosphorylation but rather by TAK1-activated IKK.

### TAK1 enhances IAV-induced cell death through RIPK1

**Fig. 5:**
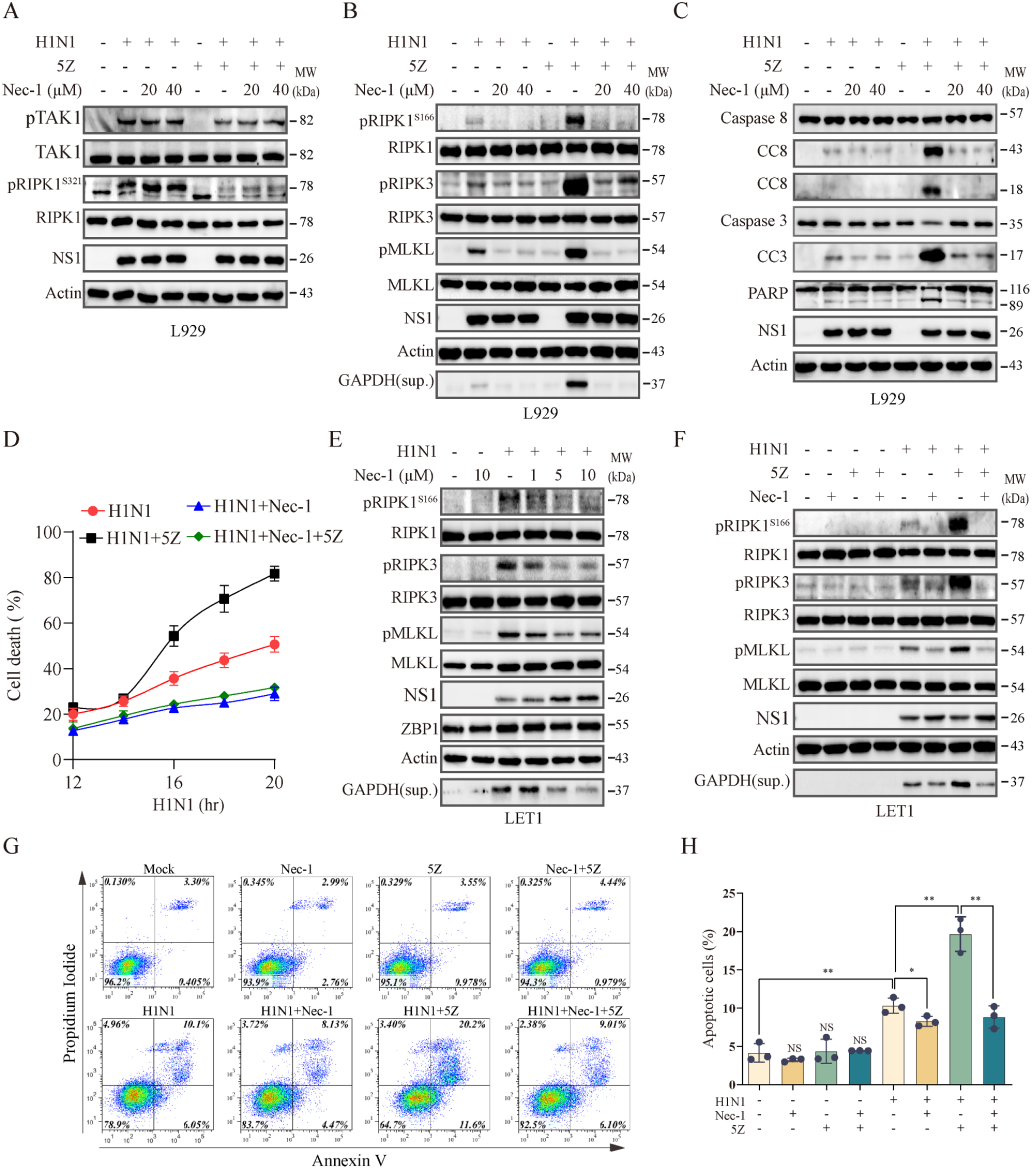
Nec-1 inhibits IAV-induced cell death in the absence or presence of TAK1 suppression. L929 cells were infected with H1N1 viruses (2 MOI) and then incubated in the absence or presence of Nec-1 (20 or 40 µM) for 12 h. 5Z (0.5 µM) was added and then incubated for another 6 h. Cell lysates were analyzed for the levels of TAK1 and RIPK1^S321^ phosphorylation (**A**), necroptosis-related protein phosphorylation (RIPK1^S166^, RIPK3, and MLKL), actin, and GAPDH in the conditioned media (**B**), and apoptosis-related proteins (**C**) by Western blot. (**D**) L929 cells seeded in a 96-well plate were infected with 2 MOI of H1N1 and then incubated in the absence or presence of Nec-1 (20 µM) and/or 5Z (0.5 µM). Cells labelled with PI (2 µg/ml) were read at the indicated time in a plate reader. Data represents the mean ± SD of the triplicate from one of three independent experiments with similar results. (**E**) LET1 cells were infected with H1N1 viruses (2 MOI). Nec-1 was added at the indicated concentrations for 18 h. Cell lysates were analyzed for the levels of RIPK1, RIPK3, and MLKL phosphorylation and their corresponding total protein, ZBP1, NS1 and β-actin by Western blot. GAPDH in the conditioned media was also probed. (**F**) LET1 cells were infected with H1N1 viruses (2 MOI) and then incubated in the absence or presence of Nec-1 (10 µM) for 12 h. 5Z (0.5 µM) was added and then incubated for another 12 h. Cell lysates were analyzed for the levels of RIPK1, RIPK3, and MLKL phosphorylation and their corresponding total protein as well as NS1 and β-actin by Western blot. GAPDH in the conditioned media was also probed. (**G** & **H**) L929 cells infected with 1 MOI of H1N1 viruses were treated with 5Z (0.5 µM) minus or plus Nec-1 (20 µM) as above. Single-cell suspensions were stained for apoptosis with annexin V plus propidium iodide (**G** & **H**). The results represent the mean percent of cell death ± SD of three independent experiments. ^*^p < 0.05, ^**^p < 0.01

Having shown that TAK1 inhibition enhanced RIPK1^S166^ phosphorylation and activation and accelerated IAV-induced cell death, we next tested if RIPK1 indeed was required for IAV-induced cell death in the absence or presence of TAK1 inhibition. Nec-1, an inhibitor of RIPK1, slightly increased RIPK1^S321^ phosphorylation in the absence or presence of 5Z but did not affect TAK1 phosphorylation in H1N1-infected L929 cells (Fig. 5A). Nec-1 decreased IAV-induced RIPK1^S166^, RIPK3, and MLKL phosphorylation and inhibited GAPDH release into the conditioned media of H1N1 virus-infected L929 cells in the absence or presence of 5Z (Fig. 5B). Nec-1 slightly inhibited IAV-induced caspase-3, caspase-8, and PARP cleavage in the absence of 5Z. 5Z dramatically increased IAV-induced caspase-3, caspase-8, and PARP cleavage in L929 cells, which was largely blocked by Nec-1 (Fig. 5C). 5Z significantly increased IAV-induced cell death (Fig. 5D). Nec-1 blocked IAV-induced L929 cell death in the absence or presence of 5Z (Fig. 5D). We also investigated the role of RIPK1 in IAV-induced necroptosis in LET1 cells. Nec-1 inhibited H1N1 virus-induced RIPK1^S166^, RIPK3, MLKL phosphorylation modestly in dose-dependent manner in LET1 cells and inhibited H1N1 virus-induced GAPDH release into the conditioned media of virus-infected cells (Fig. 5E). 5Z dramatically increased H1N1 virus-induced RIPK1^S166^, RIPK3, MLKL phosphorylation, which was abrogated by Nec-1 (Fig. 5F).

We then assessed the effect of TAK1 and RIPK1 inhibition on IAV-induced cell death. Flow cytometric analysis of L929 cells stained with annexin V and propidium iodide revealed that 5Z and Nec-1 alone or combination did not affect cell death in mock-infected L929 cells (Fig. 5G & H). H1N1 virus significantly increased the number of dead cells, which was slightly inhibited by Nec-1 (Fig. 5G & H). 5Z dramatically enhanced IAV-induced cell death in L929 cells, which was also largely blocked by Nec-1 (Fig. 5G & H). Of note, IFN-g can induce phosphatidylserine exposure in a MLKL-dependent but caspase-8-independent manner [41], and phosphatidylserine externalization also occurs in necroptotic cells [42], great cautions should be taken when interpreting annexin V- and PI-positive cells as apoptotic.

### RIPK1 is required for IAV-induced apoptosis and necroptosis

**Fig. 6:**
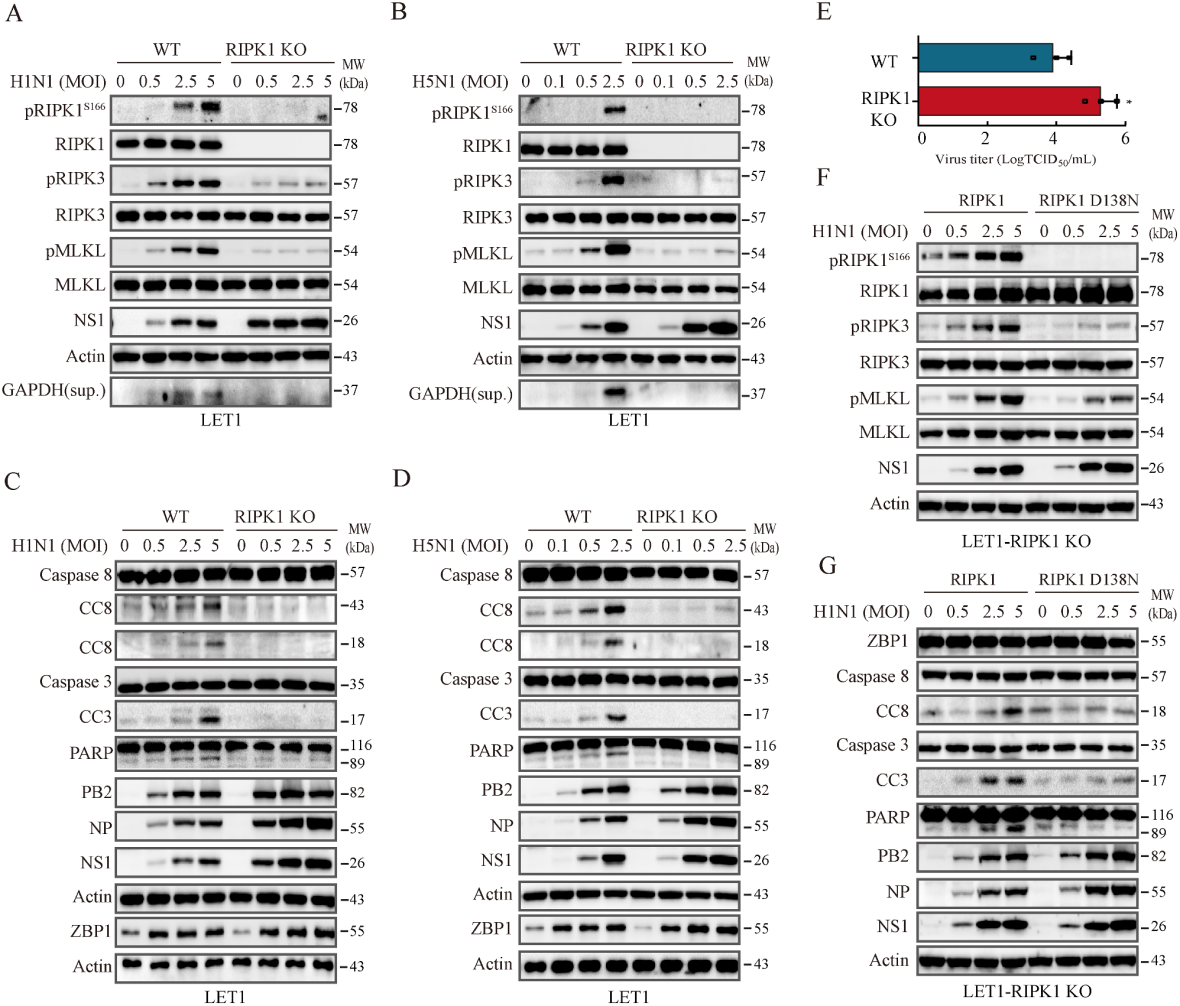
RIPK1 deficiency ameliorates IAV-induced cell death. Control and RIPK1 knockout LET1 cells were infected with the indicated MOI of H1N1 (**A** & **C**) or H5N1 (**B** & **D**) viruses and incubated for 18 h. Cell lysates were analyzed for the levels of necroptosis-related protein phosphorylation (RIPK1^S166^, RIPK3, and MLKL), NS1, actin, and GAPDH in the conditioned media (**A** & **B**), and apoptosis-related proteins (**C** & **D**) by Western blot. (**E**) RIPK1 knockout enhances virus replication. Control and RIPK1 knockout LET1 cells were infected with 0.5 MOI of H5N1 virus. After incubation for 18 h, the virus titers in the conditioned media were measured by measuring the TCID50 values. Data represents the mean ± SD of three independent experiments. Unpaired Student’s t test was used to determine the difference between WT and RIPK1-knockout cells infected H5N1 virus.   *p＜0.05.  (**F** & **G**) RIPK1 knockout LET1 cells were first transfected with a murine wild-type RIPK1 or a kinase-dead (KD) mutant RIPK1 gene (D138N). After incubation for 30 h, the cells were infected with the indicated MOI of H1N1 viruses and then incubated for another 18 h. Cell lysates were analyzed for necroptosis- and apoptosis-related proteins by Western blot

Balachandran and colleagues reported that IAV infection induces necroptosis in MEFs in a RIPK1-independent manner, and that IAV-induced apoptosis does not require RIPK1 activity [14, 15]. To confirm the role of RIPK1 in IAV-induced apoptosis and necroptosis, we generated RIPK1-knockout LET1 cells to determine if RIPK1 deficiency blocked IAV-induced cell death. RIPK1 knockout dramatically reduced H1N1- and H5N1 virus-induced RIPK3 and MLKL phosphorylation and abrogated the release of GAPDH into the conditioned media of virus-infected LET1 cells (Fig. 6A & B). Of note, a small fraction of RIPK3 and MLKL could still become phosphorylated in RIPK1 knockout cells infected with IAV. This could be attributed to the direct activation of RIPK3 by ZBP1 following influenza virus infection. RIPK1 knockout in LET1 cells also prevented H1N1 and H5N1 virus-induced cleavage of caspase-8, caspase-3 and PARP (Fig. 6C & D). However, RIPK1 knockout in LET1 cells increased the levels of NS1, PB2, and NP proteins (Fig. 6A-D) and H5N1 virus titers (Fig. 6E). RIPK1 knockout did not significantly change the level of ZBP1 expression in the clone used here in the absence or presence of H1N1 virus infection at multiple time points (Fig. 6B & Fig. S2A). In some RIPK1-deficient clones, ZBP1 levels were slightly or moderately decreased, compared to their Lenti-V2 control cells. However, ZBP1 overexpression did not restore the ability of H1N1 virus-induced RIPK3 and MLKL phosphorylation nor caspase-8 cleavage (Fig. S2B & C).

To confirm that RIPK1 enzymatic activity was indeed required for IAV-induced cell death, we tested if the expression of a kinase-dead (KD) RIPK1 gene (D138N mutant) in RIPK1-defcient LET1 cells was able to restore the ability of IAV to induce cell death. H1N1 virus induced the phosphorylation of RIPK1^S166^, RIPK3, and MLKL (Fig. 6F), and cleavage of caspase-8, caspase-3, and PARP (Fig. 6G) in RIPK1 knockout LET1 cells transfected with wild-type RIPK1 but did a little in the cells transfected with the KD-RIPK1 gene. These observations collectively suggest that IAV-induced necroptosis largely requires RIPK1 kinase activity, IAV may also induce necroptosis via the ZBP1-RIPK3-MLKL pathway.

### ZBP1 is required for IAV-induced apoptosis and necroptosis, whereas RIPK3 is required for IAV-induced necroptosis but not apoptosis

**Fig. 7:**
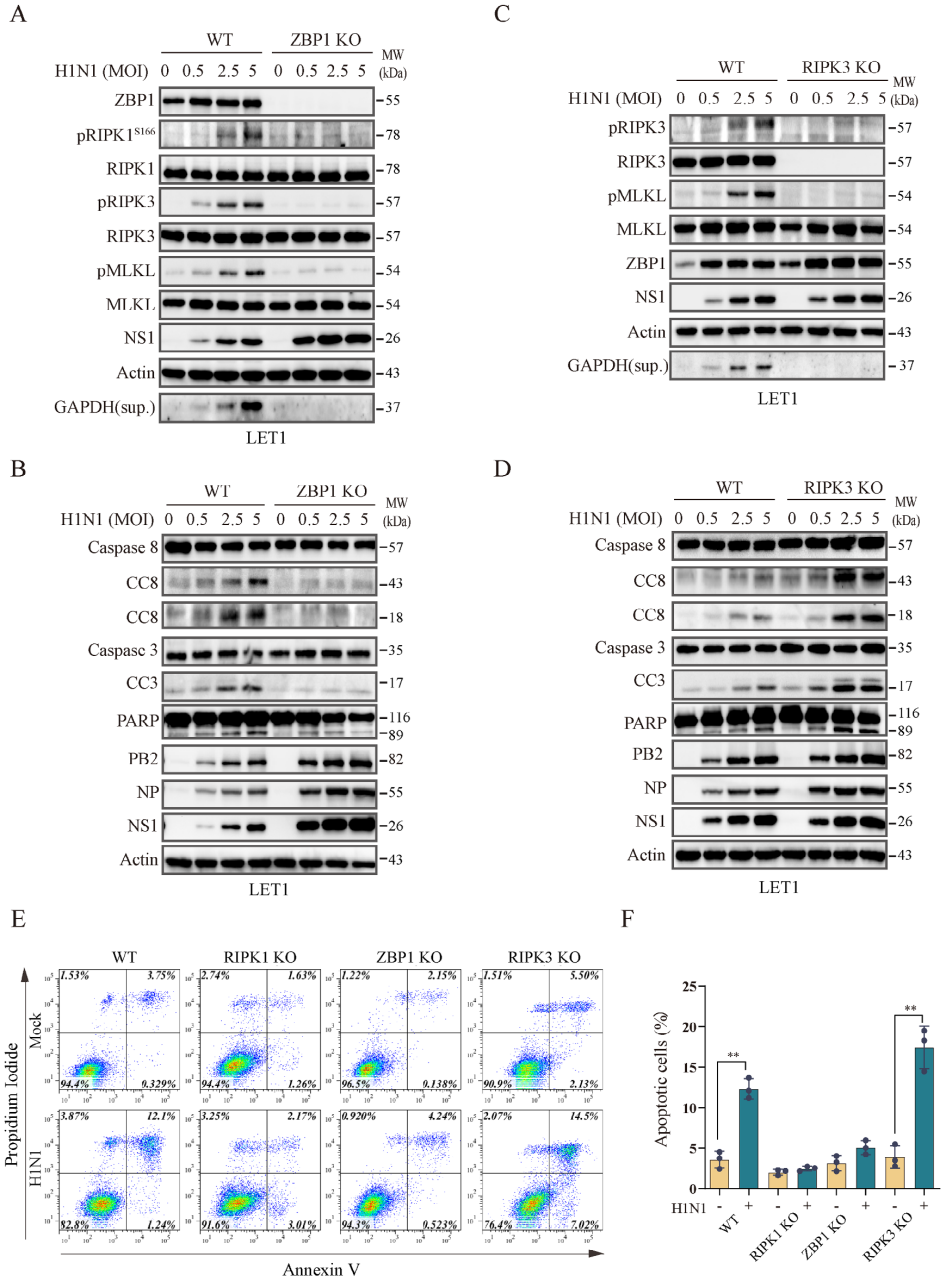
Role of ZBP1 and RIPK3 in IAV-induced cell death. Control and ZBP1 (**A** & **B**) or RIPK3 (**C** & **D**) knockout LET1 cells were infected with the indicated MOI of H1N1 viruses and incubated for 18 h. Cell lysates were analyzed for the levels of necroptosis-related protein phosphorylation (RIPK1^S166^, RIPK3, and MLKL), actin, and GAPDH in the conditioned media (**A** & **C)**), and apoptosis-related proteins (**B** & **D**) by Western blot. (**E** & **F**) Role of RIPK1, RIPK3, and ZBP1 deficiency in IAV-induced cell death. Control and RIPK1, RIPK3, or ZBP1 knockout LET1 cells were infected with 1 MOI of H1N1 viruses and incubated for 24 h. Single-cell suspensions were stained for apoptosis with annexin V plus propidium iodide (**E** & **F**). The results represent the mean percent of cell death ± SD of three independent experiments. ^*^p < 0.05, ^**^p < 0.01

Both ZBP1 and RIPK3 have been implicated in playing a crucial role in IAV-induced necroptosis and apoptosis in MEFs [43–45]. We tested if they were also required for IAV-induced cell death in LET1 cells. ZBP1 deficiency abrogated H1N1 virus-induced RIPK1^S166^, RIPK3, and MLKL phosphorylation and GAPDH release in the conditioned media of virus-infected LET1 cells (Fig. 7A). ZBP1 deficiency also prevented H1N1 virus-induced caspase-8, caspase-3, and PARP cleavage (Fig. 7B). Similar observations were made in ZBP1 knockout LET1 cells infected with H5N1 virus (Fig. S3). We next tested if RIPK3 was also required for IAV-induced cell death in LET1 cells. RIPK3 knockout abrogated MLKL phosphorylation and the release of GAPDH into the conditioned media of H1N1 virus-infected LET1 cells (Fig. 7C). In contrast, RIPK3 knockout did not abrogate but rather increased IAV-induced caspase-8, caspase-3, and PARP cleavage, probably due to slightly increased virus replication (Fig. 7D). This finding is opposite to the previous observations made in MEFs that RIPK3 is required for IAV-induced apoptosis [14, 15].

Finally, we confirmed the role of ZBP1, RIPK1, and RIPK3 in IAV induced-cell death. Annexin V plus PI staining revealed that LET1 cells infected with IAV undergone late apoptosis, as evidenced by the presence of a population in the upper-right quadrant. Of note, very few cells infected with H1N1 virus were in the stage of early apoptosis, which is consistent with the observation made in the nasal mucosal epithelial cells in a recent publication [46]. Both ZBP1 and RIPK1 deficiency blocked IAV-induced cell death effectively (Fig. 7E & F). In contrast, RIPK3 knockout did not inhibit H1N1 virus-induced apoptosis but slightly increased the number of cells undergoing the early apoptosis (lower-right quadrant) (Fig. 7E & F).

### Inhibition of TAK1 activity by 5Z enhances IAV-cell death in vivo

Next, we investigated whether IAV infection also induced cell death in the lungs of virus-infected mice. The levels of TAK1 and RIPK1^S321^ phosphorylation, as well as their total proteins, were elevated in the lung tissues of H1N1 virus-infected mice on day 5 post-infection, compared to that in mock-infected mice (Fig. S4A). The levels of RIPK1^S166^, RIPK3, and MLKL phosphorylation and total RIPK1 were significantly higher in the lung tissues of H1N1 virus-infected mice than in the uninfected controls (Fig. S4B). Elevated levels of total caspase-8, caspase-3, RIPK1, and RIPK3 proteins in the lung tissues of IAV-infected mice are consistent with the observations made in several prior studies [47–49]. Of note, once MLKL was phosphorylated, it mobilized slightly slower during electrophoresis. This did not occur in cell culture, though the same specific anti-MLKL antibody was used for detecting MLKL in cell lines and lung tissues. The levels of cleaved and full-length caspase-8, caspase-3, and PARP proteins were significantly higher in the lung tissues of H1N1 virus-infected mice than that in control mice (Fig. S4C).

**Fig. 8:**
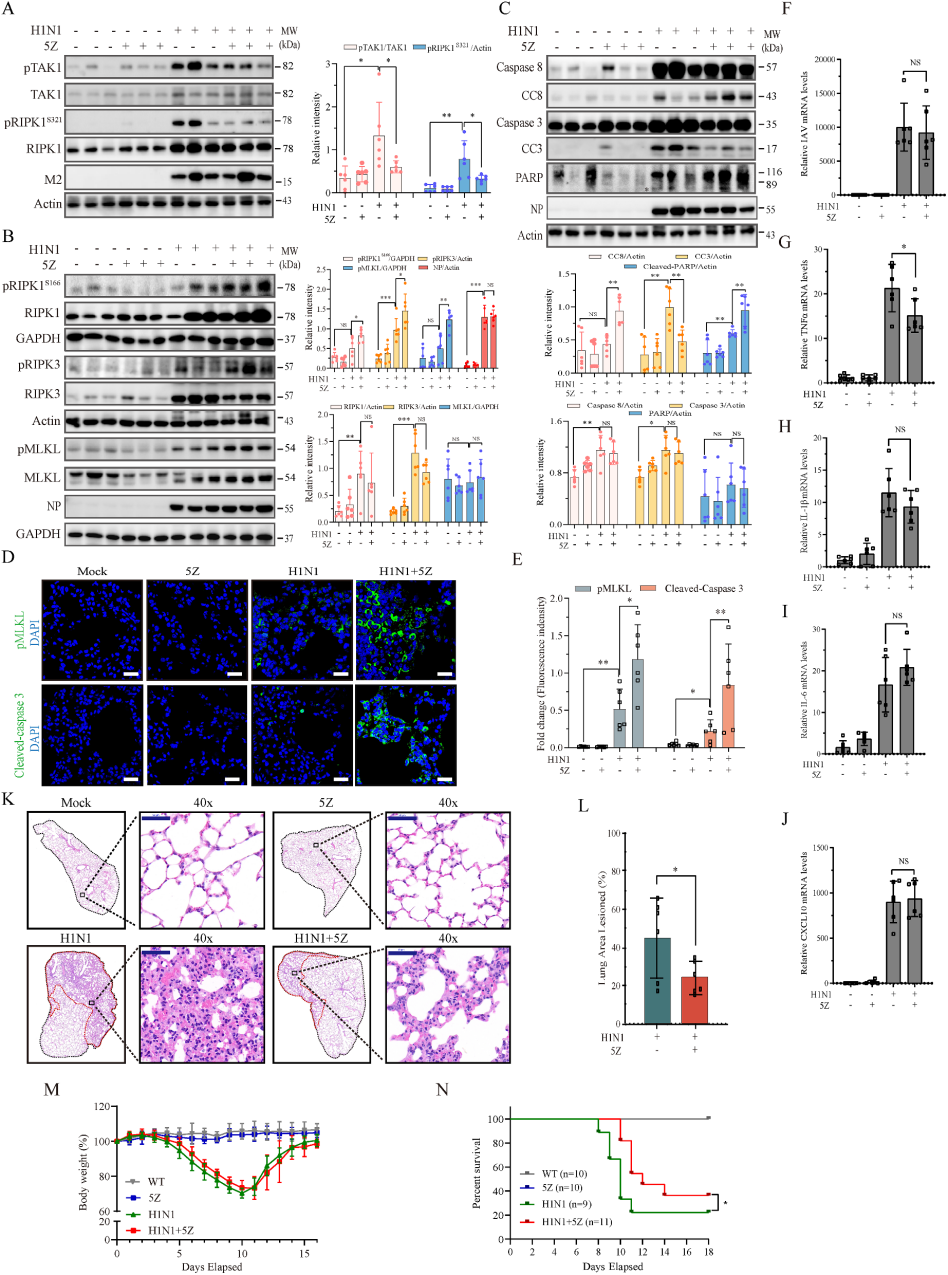
5Z enhances IAV-induced cell death in vivo. Female C57BL/6J mice (6-8-wks-old, 6 mice/group) were mock-infected or infected with H1N1 viruses (1000 pfu/mouse). Two days later, mice were treated daily with vehicle or 5Z (2 mg/kg body weight) for 3 days. On 5th days post infection, mice were given a last dose at the same dosage of 5Z 8 h prior to sacrifice. Lung tissues were collected and analyzed for the levels of TAK1 and RIPK1^S321^ phosphorylation (**A**), necroptosis-related protein phosphorylation (RIPK1^S166^, RIPK3, and MLKL) and β-actin (**B**), and apoptosis-related proteins (**C**) by Western blot. The density of the bands from 6 mice per group was analyzed using NIH Image-J software and normalized by the arbitrary units of their total protein bands or β-actin or GAPDH. ^*^p < 0.05, ^**^p < 0.01, ^***^p < 0.001. (**D & E**) 5Z increases H1N1 virus-induced cell death. The sections of the paraffin-embedded lung tissue blocks were stained with antibodies against phosphorylated MLKL or cleaved caspase-3 as described in Methods. The fluorescence intensity of phosphorylated MLKL or cleaved caspase-3 was quantified and plotted as a bar graph. Scale bar, 20 μm. The results represent the mean ± SD of 5 random fields per section per mouse, from 6 mice per group. ^*^p < 0.05, ^**^p < 0.01. (**F**-**L**) Female C57BL/6J (6-8-week- old, 6 mice/group) were intranasally mock-infected PBS or infected with H1N1 virus (1000 pfu) and then treated daily with the vehicle or 5Z (2 mg/kg body weight) for 5 days. On 4th days post infection, mice were given a last dose at the same dosage of 5Z 8 h prior to sacrifice. Lung tissues were collected and analyzed for the levels of viral mRNA (**F**) and inflammatory cytokines (**G-J**) by RT-PCR or inflammation by H & E staining (**K **& **L**). Scale bar, 50 μm. Data represents the mean ± SD of the lung tissues from six animals. *p＜0.05. (**M **& **N**) Female C57BL/6J mice (6–8 weeks old, 9–11 mice/group) were mock-infected with PBS or infected intranasally with H1N1 viruses (1000 pfu/mouse) and then treated daily with the vehicle or 5Z (2 mg/kg body weight) for 7 days. Mice were weighed and monitored for survival for 16 days. Percent of bodyweight changes (**M**) and percent survival (**N**) were plotted. *p < 0.05, compared to the untreated controls

We then investigated the impact of TAK1 inhibition on IAV-induced cell death in the mouse lung tissue. IAV infection increased the level of total RIPK1, RIPK3, caspase-8, and PARP proteins (Fig. 8A-C). This is likely due to the up-regulation of gene expression by cytokines such as IFNs, which readily induce caspase-8 expression [50]. In addition, the infiltration of immune cells may also lead to increased levels of these proteins. 5Z treatment (2 mg/kg bodyweight) for 5 days lowered the levels of TAK1 and RIPK1^S321^ phosphorylation but did not significantly decrease the levels of their total proteins in the lung tissues of H1N1 virus-infected mice, compared to that in H1N1 virus-infected mice treated with the vehicle control (Fig. 8A). 5Z treatment further increased the levels of RIPK1^S166^, RIPK3, and MLKL phosphorylation but did not significantly affect total RIPK1 and RIPK3 proteins in the lung tissues of H1N1 virus-infected mice (Fig. 8B). 5Z treatment further increased the cleavage of caspase-8 and PARP proteins but unexpectedly decreased caspase-3 cleavage in the lung tissues of H1N1 virus-infected mice (Fig. 8C). 5Z treatment did not significantly affect the levels of RIPK1^S321^, RIPK1^S166^, TAK1, RIPK3, and MLKL phosphorylation or their total proteins and neither affected caspase-3, caspase-8, and PARP cleavage in the lung tissues of mock-infected mice (Fig. 8A-C). The levels of RIPK1^S166^, RIPK3 and MLKL phosphorylation and the levels of caspase-8, caspase-3, and PARP cleavage in the lung tissues of H1N1 virus-infected mice treated with 5Z for 3 days were also significantly higher than those treated with the vehicle (Fig. S5A & B). Of note, there is inconsistency in caspase-3 activation analyzed by Western blot and immunofluorescent staining. This is likely because the former detects caspase-3 cleavage in the lysates of entire lungs, whereas the latter detects caspase-3 activation specifically in IAV-infected alveolar epithelial cells. 5Z treatment did not affect the levels of the viral M2 and NP proteins (Fig. 8A-C). Immunofluorescence staining revealed that 5Z treatment significantly increased the number of alveolar epithelial cells that stained positive for phosphorylated MLKL and cleaved caspase-3 (Fig. 8D & E). These observations suggest that 5Z treatment also activates RIPK1 in vivo and enhances IAV-induced apoptosis and necroptosis in vivo in the lungs of IAV-infected mice.

### The anti-inflammatory and therapeutic effects of 5Z treatment

Cell death is implicated in playing important roles in the inflammatory response and in virus replication. We first investigated if TAK1 inhibition affected virus replication and inflammatory cytokine production. RT-PCR analysis revealed that 5Z treatment did not affect the levels of viral mRNA of H1N1 virus in the lung tissues collected on day 5 post-infection (Fig. 8F). 5Z treatment slightly but significantly decreased the levels of TNF-α but not IL-1β, IL-6, and CXCL10 mRNAs in the lung tissues of H1N1 virus-infected mice (Fig. 8G-J). Hematoxylin & eosin (H&E) staining revealed that inflammation was slightly but significantly lower in the lungs of H1N1 virus-infected mice treated with 5Z than the control vehicle (Fig. 8K & L). We then determined if 5Z treatment had any therapeutic effect. As shown in Figs. 8M & 5Z treatment did not affect the change of bodyweight of H1N1 virus-infected mice and did not affect the bodyweight of uninfected mice either. 5Z treatment slightly but significantly prolonged the survival of H1N1 virus-infected mice (Fig. 8N). These observations suggest that 5Z treatment has a very weak anti-inflammatory and therapeutic effect on IAV infections.

## Discussion

**Fig. 9:**
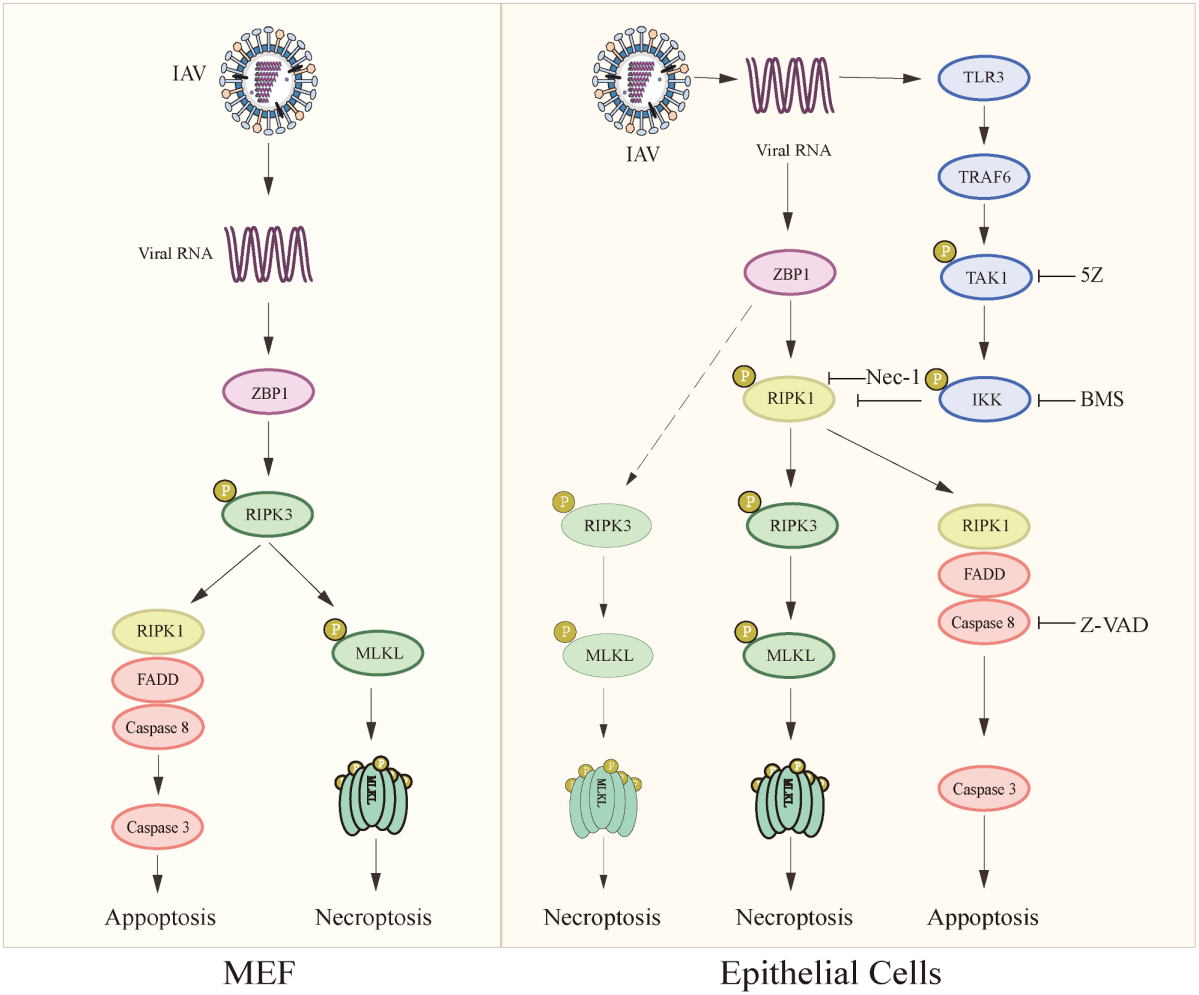
The schematic model of IAV-induced cell death. Viral RNA of IAV binds to ZBP1 and induces RIPK1 phosphorylation and activation. Activated RIPK1 interacts with FADD and caspase-8 to induce apoptosis. RIPK1 binds to and activates RIPK3, which then phosphorylates and activates MLKL to induce necroptosis. ZBP1 can bypass RIPK1 and directly engage and activate RIPK3 to induce necroptosis through MLKL. TAK1 is activated by viral RNA through TLR3. TAK1 suppresses RIPK1 activation through IKK-mediated phosphorylation of RIPK1 at S25. TAK1 represses RIPK1 activation and RIPK1-dependent apoptosis and necroptosis to facilitate virus replication. IAV induces cell death in MEFs and non-MEFs by two different pathways

Programmed cell death is thought to be the first-line defense of host cells to restrict virus replication [8, 30, 31]. Influenza virus is a fast-replicating RNA virus that induces apoptosis and necroptosis in fibroblast and epithelial cells [8]. However, whether IAV infection can suppress or delay cell death to facilitate its replication remains elusive. Our present study shows that IAV infection activated TAK1 to suppress RIPK1 activation and attenuate RIPK1-depedent apoptosis and necroptosis. Our study uncovers a previously unrecognized role of TAK1 in repressing virus-induced cell death and suggests that IAV-induced necroptosis requires both RIPK1 and RIPK3, whereas IAV-induced apoptosis requires RIPK1 activity but not RIPK3. These findings are in sharp contrast to the observations made in MEFs that IAV-induced necroptosis requires RIPK3 but not RIPK1, and IAV-induced apoptosis requires RIPK3 and RIPK1 but not RIPK1 activity [14, 15] (Fig. 9).

TAK1 activation suppresses RIPK1 activity in various settings [51–54]. TAK1 blocks TNF-α-induced RIPK1 activation and apoptosis in melanoma [51] and LPS-induced necroptosis in bone marrow-derived macrophages [51]. TAK1 directly phosphorylates RIPK1^S321^ in MEFs and bone marrow-derived macrophages [24] or by its downstream p38-MK2 kinases [24–27, 29]. TAK1 also activates IKK to phosphorylate RIPK1^S25^ and suppress RIPK1 activation [28]. Our present study shows that the TAK1 inhibitor 5Z and TAK1 knockout blocked IAV-induced RIPK1^S321^ phosphorylation but increased RIPK1^S166^ phosphorylation in L929 and LET1 cells. Although the inhibitors of p38 and MK2 did block IAV-induced RIPK1^S321^ phosphorylation but did not increase IAV-induced RIPK1^S166^ phosphorylation. In contrast, the IKK inhibitor BMS345541 increased IAV-induced RIPK1^S166^ phosphorylation. These observations suggest that TAK1 suppresses RIPK1 activation and IAV-induced cell death through IKK but not by p38-activated MK2. Of note, great cautions should be taken in making this conclusion since the IKK and p38-MK2 pathways were only blocked by using their inhibitors, which may have some unknown off-target effects.

Several prior studies have shown that ZBP1 can directly bind and activate RIPK3 to phosphorylate MLKL^S345^ to induce necroptosis, RIPK1 is not required for IAV-induced necroptosis in MEFs [14, 15, 55]. In contrast, our present study shows that RIPK1 inhibition by Nec-1 or knockout inhibited IAV-induced RIPK1^S166^ phosphorylation in L929 cells and LET1 cells and abrogated IAV-induced necroptosis when TAK1 was inhibited. Consistent with these observations, Nec-1 treatment decreases epithelial necrosis and bronchial degeneration within the airway of cIAP-deficient mice [47] and inhibits H1N1 virus-induced necroptosis in human THP1 macrophages [56]. Based on these observations, we propose that IAV infection induces necroptosis largely by the ZBP1-RIPK1-RIPK3-MLKL complex in LET1, with a minor contribution from the ZBP1-RIPK3-MLKL pathway (Fig. 9). We attempted to dissect why IAV-induced necroptosis pathways are different in MEFs and non-MEFs. ZBP1 was expressed at much higher levels in MEFs than in L929 (Fig. S6A). IAV only slightly induced RIPK1^S166^ phosphorylation in MEFs, and Nec-1 had no effect on H1N1 virus-induced MLKL phosphorylation (Fig. S6B). Caspase-6 enhances the interaction between RIPK3 and ZBP1 [57, 58]. Sperm-Associated Antigen 9 (SPAG9), another ZBP1-interacting protein, can be phosphorylated by JNK and enhances IAV-induced cell death [59]. Thus, differential levels of ZBP1, caspase-6, and SPAG9 expression in different cell types may lead to different IAV-induced necroptosis pathways.

Previous studies have shown that IAV-induced apoptosis in MEFs does not require RIPK1 activity [14, 15], and that IAV induces apoptosis via the ZBP1-RIPK3-RIPK1-FADD-caspase-8 complex in MEFs [15] (Fig. 9). In contrast, we found that inhibition of RIPK1 by Nec-1 and knockout suppressed IAV-apoptosis. Re-expression of wild-type RIPK1 in RIPK1-defient cells restored IAV-induced cell death more effectively than that transfected with kinase-dead RIPK1. Consistent with this observation, RIPK1 kinase activity is required for spontaneous cell death in TAK1-deficient macrophages [60]. We propose that RIPK1 regulates IAV-induced apoptosis by the ZBP1-RIPK1-FADD-caspase-8 complex, and that RIPK3 is not required for IAV-induced apoptosis (Fig. 9).

Viruses develop various strategies to manipulate cell death to keep the “virus factory” functional [30, 31]. ZBP1 or RIPK3 deficiency dramatically increases IAV replication in vitro in LET1 cells and in vivo in the lung tissues of H1N1 virus-infected mice [14, 15, 34, 61]. Consistently, our study shows that IAV replicated much faster in RIPK1, RIPK3, and ZBP1-deficient cells than in wild-type cells. Since TAK1 activation plays a dominant role in suppressing both apoptosis and necroptosis, inhibition of TAK1 by 5Z or gene knockout suppressed IAV replication (Fig. S7) [62]. In contrast, 5Z treatment enhanced apoptosis and necroptosis in the lungs of IAV-infected mice but did not affect IAV replication on day 5 and only slightly improved the survival of IAV-infected mice. Due to the complex roles of TAK1 in inflammation [19], autophagy [40], and the integrity of alveolar barriers [63], the antiviral activity observed in vitro could not be replicated in vivo. Furthermore, the viral dose used to infect mice may also affect whether cell death impacts the survival of mice [55, 64]. Intriguingly, several recent studies showed that inhibition of apoptosis or necroptosis does not enhance virus replication but rather ameliorates viral pathogenesis in several settings. For example, Chu et al. [65] reported that inhibition of Middle East respiratory coronavirus (MERS-CoV)-induced apoptosis by the protein kinase R-like endoplasmic reticulum kinase (PERK) inhibitor prevents lung damage in a mouse model; Treatment with the intrinsic apoptosis inhibitor z-LEHD-fmk rescues the survival of mice infected with MERS-CoV or SARS-CoV. Wang et al. [66] reported that inhibition of PERK-induced apoptosis by TRIM29 deficiency or by the PERK inhibitor GSK2656157 alleviates myocarditis and prolongs the survival of coxsackievirus B3 (CVB3)-infected mice. Gautam et al. [67] recently reported that inhibition of necroptosis by UH15-38, a RIPK3-specific inhibitor, blocks IAV-induced lung injury in a severe influenza mouse model. These observations further point out the complex effects of targeting virus-induced cell death on virus replication, antiviral immunity, and inflammatory responses Investigation with lung tissue-specific conditional TAK1 knockout mice should provide definitive evidence if inhibition of cell death by TAK1 activation enhances IAV replication.

In summary, our study provides evidence that TAK1 activation prevents premature cell death and enhances IAV replication; both RIPK1 and its enzymatic activity are required for IAV-induced apoptosis and necroptosis, whereas RIPK3 is required for IAV-induced necroptosis but not apoptosis. Our study indicates that IAV induces cell death in airway epithelial cells via a pathway that is different from that in MEFs.

## Materials and methods

### Reagents and antibodies

5Z-oxzeneonal was purchased from Cayman Chemical (Ann Arbor, MI, USA) and MedChemExpress (Monmouth Junction, NJ, USA). Necrostatin-1 (Nec-1) was purchased from Selleck Inc. (Shanghai, China). BMS345541 was purchased from Tocris Bioscience (Bristol, UK). PF-3644022, z-VAD(OMe)-FMK, AT-406 (SMAC) were purchased from MedChemExpress (Monmouth Junction, NJ, USA). Recombinant mouse TNF-α was purchased from R&D Systems (Minneapolis, MN, USA). DAPI (4’,6-Diamidino-2-Phenylindole, Dilactate) was purchased from Invitrogen (Grand Island, NY, USA). TurboFect Transfection Reagent, FastDigest KpnI and FastDigest NheI were purchased from Thermo Scientific (Waltham, MA, USA). FITC Annexin V Apoptosis Detection Kit I was purchased from BD Pharmingen ((San Jose, CA, USA). pEASY^®^-Uni Seamless Cloning and Assembly Kit was purchased from transgen (Beijing, China). PrimeScriptTMRT Master Mix and TB Green^®^ Premix Ex TaqTM II were purchased from TaKaRa (Dalian, China). Antibodies for TNFR1 (223352), and RIPK3 (phospho-T231 + S232) (222320 were purchased from Abcam (Cambridge, MA, USA). Antibodies for MLKL (37705), mRIPK3 (95702), mRIPK1 (phospho-S166) (53286), mRIPK1 (phospho-S321) (83613), anti-RIPK1 (3493), mouse-cleaved caspase-8 (Asp387) (8592), anti-caspase-8 (4790), cleaved caspase-3 (9664), caspase-3 (14,220), PARP (9532), TAK1 (phospho-Thr187) (4536), TAK1 (4505), p38 (phospho-Thr180/Tyr182) (4511), p38 (9212), IKKα/β (phospho-S176/180) (2697) and IKKα (2682) were purchased from Cell Signaling Technology (Danvers, MA, USA). Anti-Influenza A virus PB2 (125926) and anti-Influenza A virus NP (125989) were purchased from GeneTex Inc. (Irvine, CA, USA). Anti-Influenza A virus NS1 (130568), anti-Influenza A virus M1 (FluAc) (69824), anti-Influenza A virus M2 (32238), β-Actin (47778), and GAPDH (166574) antibodies were purchased from Santa Cruz Biotechnology, Inc. (Santa Cruz, CA, USA). Anti-ZBP1 antibody (AG-20B-0010) was purchased from AdipoGen (San Diego, CA, USA). Alexa Fluor$\text{\circledR }$488 anti-Rabbit IgG (121665) was purchased from Jackson ImmunoResearch (West Grove, PA, USA). Rat IgG1 isotype control (MAB005) and Rat anti-mouse TNF-α antibody (MAB4101) were purchased from R&D Systems (Minneapolis, MN, USA).

### Cell culture

LET1 cells, a murine lung epithelial type I cell line, were kindly provided by BEI Resources (Manassas, VA). L929 and LET1 cells were grown in DMEM containing 10% fetal bovine serum (FBS). MEFs were prepared by trypsin digestion of 13-day embryos of C57BL/6J mice. The 1st -3rd passages of monolayers were used for virus infection. A/mallard/Huadong/S/2005 (SY strain, H5N1) [68–70]. A/PR8/34 (H1N1) virus was kindly provided by Dr. Liqian Zhu (College of Veterinary Medicine, Yangzhou University). These viruses were prepared by inoculating 10-day-old specific-pathogen-free embryonic chicken eggs. The virus titers were determined by a 10-fold serial dilution (10^1^ to 10^9^, dilutions (10^5^ − 10^9^) were used to infect MDCK. The 50% tissue culture infection dose (TCID_50_/100 µl) values were determined by using the standard Reed and Muench method.

### Gene knockout

RIPK1, ZBP1, TNFR1, RIPK3 and TAK1 knockout were carried out by transfecting LET1 cells with a CRISPR expression vector encoding a guild RNA that targets RIPK1, ZBP1, TNFR1, RIPK3, and TAK1. The targeting sequences were as follows: mRIPK1, 5’-AGACAGCGGAGGCTTCGGGA-3’; mZBP1, 5’-CAGGTGTTGAGCGATGACGG-3’; mTNFR1, 5’-GCAG-CAGGCCAGGCACGGTG-3’; mRIPK3, 5’-GGAACCGCTGACGCACCAGT-3’ and 5’-TTCAGGGAGGGTCCCAGTCA-3’; mTAK1, 5’-GATGATCGAAGCGCCGTCGC-3’ and 5’-TCGAAGTTCAGGACCTGCGA-3’. The complementary oligonucleotides containing 4 extra nucleotides at each end were annealed and ligated to the Bmsb I-digested LentiCRISPRv2 vector with the T4 DNA ligase. LET1 cells seeded in a 24-well plate were transfected with the LentiCRISPRv2 vector or the vector encoding sgRNA by using the TurboFect transfection reagent following the manufacturer’s instruction. After incubation for 6 h, culture media were replaced with fresh complete media. Forty hours later, single cell suspensions were prepared, seeded in 6-well plates, and grown in the media containing puromycin (3 µg/ml). Fresh media containing the same concentrations of puromycin were changed every three days. After culturing for 3 weeks, individual clones were picked, expanded and analyzed for RIPK1, ZBP1, TNFR1, RIPK3, and TAK1 expression by Western blot. At least two clones that lack the expression of RIPK1, ZBP1, TNFR1, RIPK3, and TAK1 were used for investigating their role in IAV-induced cell death.

### Recombinant DNA

Wild-type and kinase-dead (D138N) mRIPK1 were amplified from pBOB-mRIPK1 and pBOB-mRIPK1 D138N plasmids [71] (kindly provided by Prof. Jiahuai Han, Xiamen University, China) were cloned into the Kpn I/Nhe I-digested pCAGGS vector by using Seamless Cloning and Assembly Kit (Transgen, Beijing, China) to generate pCAGGS-mRIPK1 and pCAGGS-mRIPK1 D138N plasmids. The mZBP1 gene was synthesized by reverse transcription of total mRNA extracted from LET1 cDNA and cloned into the Kpn I/Nhe I-digested pCAGGS vector by using Seamless Cloning and Assembly Kit (Transgen, Beijing, China) to generate pCAGGS-mZBP1 plasmid.

### Western blot

Cells grown in 12-well plates were harvested and lysed in NP-40 lysis buffer (50 mM Tris-HCl (pH 8.0), 150 mM NaCl, 1% NP-40, 5 mM EDTA, and the protease inhibitor cocktail (Pierce™, Thermo Fisher Inc., Shanghai, China). Cell lysates were sonicated and centrifuged at 4ºC for 15 min. The supernatants were collected, and protein concentrations were measured using a BCA protein assay kit. After mixing with equal volumes of 2X loading buffer and incubation at 95ºC for 5 min, equal amounts of cell lysates (15–20 µg/lane) were electrophoresed and transferred to PVDF membranes. Proteins of interest were detected by Western blot with their specific antibodies, followed by horseradish peroxidase-conjugated goat anti-rabbit or anti-mouse IgG and SuperSignal Western Pico enhanced chemiluminescence substrate (Pierce Chemical Co., Rockford, IL). Antibodies for the detection of PB2 and NP proteins were diluted at 1:8000 in the antibody dilution buffer, antibodies against M1, M2, NS1, GAPDH, and β-actin were diluted 1:800, the antibody against TNFR1 was diluted at 1:400, antibodies for other proteins were diluted at 1:2000. The experiments were repeated at least three times with similar results. The density of the bands in Fig. 8 was analyzed by using NIH Image-J software (NIH, Bethesda, MD, USA) (https://imagej.nih.gov/ij/) and normalized by the arbitrary units of their corresponding total proteins or β-actin or GAPDH as indicated.

### Cell death assay

L929 and LET1 cells seeded in a 96-well plate were infected with the indicated MOI of H1N1 (PR8 strain) or H5N1 (SY strain) for 18 h or infected with 2 MOI of H1N1 virus and then incubated for the indicated lengths of time. Cells were labelled with PI (2 µg/ml) and then read (Excitation wavelength, 535 nm; Emission wavelength, 617 nm) in a plate reader. Data represent the mean ± standard deviation (SD) of three independent experiments (Fig. 1E & F) or the triplicate from one of three experiments with similar results (other figures).

### Flow cytometry

L929 cells seeded in 6-well plates were left uninfected or infected with H1N1 viruses. After incubation in the absence or presence of Nec-1 (20 µM) for 12 h, 5Z (0.5 µM) was added and then incubated for another 6 h. Single-cell suspensions were prepared and analyzed for apoptosis by staining with propidium iodide (PI) and annexin V by using a FITC Annexin V Apoptosis Detection kit following the manufacturer’s instructions. Single-cell suspensions were run in a Beckman Coulter flow cytometer (Model CyAn ADP). The fluorescence intensity was analyzed by using the FlowJo software. Annexin-positive and PI-positive cells were gated. The percentage of Annexin V- and PI-positive cells from three independent experiments were calculated and statistically analyzed by using the unpaired Student’s *t* test. Data in bar graphs are the mean ± SD of three independent experiments.

### Animals

Animal use was approved by the Institutional Animal Care and Use Committee of Yangzhou University (Approval number 202,102,006; date of approval: February 5, 2021) and carried out in accordance with the Guide for the Care and Use of Laboratory Animals by the National Research Council. C57BL/6J mice (female, 6–8 weeks) were purchased from Charles River (Beijing, China). All mice were maintained on a 12-hr light/dark cycle and housed in ventilated cages at an ambient temperature of 22ºC. Mice were fed ad libitum on a normal chow diet (NCD). 5Z stock solution was prepared by dissolving it in dimethyl sulfoxide (DMSO) (20 mg/ml) and then diluted in PBS prior to use. Mice were deprived of water 6 h before virus infection. Mice were anesthetized with an intraperitoneal injection of sodium pentobarbital administration (100 mg/kg body weight) and then infected with H1N1 virus (1000 PFU/mouse in 50 µl PBS) intranasally. Two days later, mice were randomly divided and treated daily with vehicle or 5Z (2 mg/kg body weight) by intraperitoneal injection for 3 days. The dosage of 5Z was determined according to our previous study in IAV [63] and widely available references showing that the dose of 2 mg/kg bodyweight is sufficient to control tumor growth and metastasis. On 5th day post infection, mice were given a final dose 8 h prior to sacrifice. Mice were sacrificed by cervical dislocation. One part of the lung tissue was lysed in NP-40 lysis buffer (weight/volume, 1:15) and homogenized. Cell lysates were then analyzed for the indicated proteins by Western blot. A second part of the lung tissue was fixed in 4% paraformaldehyde in PBS and embedded in paraffin within 48 h after fixation.

To determine the therapeutic effect of 5Z, mice (6-8-wks-old, 9–11 mice per group) infected with H1N1 virus as above were treated daily with 5Z (2 mg/kg body weight in 100 µL PBS) by intraperitoneal injection for 7 days. Mice were monitored daily for body weights and survival for 16 days and were humanely sacrificed by CO2 inhalation when they became moribund or when the loss of body weight decreased by > 30%.

### Histopathology

The Sect. (5 μm) of lung tissue blocks were stained with hematoxylin and eosin (H&E). The inflammatory areas in each section of whole lungs from were quantified by using CaseViewer 2.0 software (3DHistech, Budapest, Hungary).

### Real-time quantitative PCR analysis

Total RNA was extracted from the lung tissues using TRIzol reagent (Invitrogen, Grand Island, NY, USA). RNA integrity was verified by electrophoresis. Reverse transcription of RNA was performed using the PrimeScriptTMRT Master Mix (Takara, Dalian, China) according to the manufacturer’s protocol. The cDNA was subjected to quantitative real-time PCR using TB Green^®^ Premix Ex TaqTM II (Takara, Dalian, China). The sequences of primers were listed in the supplementary Table 1. Amplification of mouse actin was included as a control. All expression levels were normalized to the β-actin levels in the same sample. Fold change was calculated by the ΔΔCT method. Percent expression was calculated as the ratio of the normalized value of each sample to that of the corresponding untreated control sample. All Real-Time RT-PCR analyses were performed in triplicate.

### Immunofluorescence staining

Lung tissues were fixed in 4% paraformaldehyde in PBS and embedded in paraffin within 48 hr after fixation. The sections of paraffin-embedded tissue blocks were dehydrated and rehydrated as described previously [72]. The sections were incubated with 5% bovine serum albumin (BSA) blocking buffer in PBS for 30 min at room temperature and then stained with anti-phospho-MLKL (1:200) and anti-cleaved caspase 3 rabbit mAb (1:400), followed by staining with Alex488-conjugated anti-rabbit IgG (1:100), respectively, for 1 hr at room temperature. Finally, the sections were stained with 10 µΜ DAPI (4’,6-diamidino-2-phenylindole) for 5 min. After washing the cells in PBS, fluorescent images were captured under a Nikon fluorescence microscope.

### Statistical analysis

The differences in Western blot band density in the lung tissues from uninfected or IAV-infected mice were statistically analyzed by using an unpaired Student *t* test. The differences in the areas of inflammation, percent of dead cells, percent of MLKL-positive or cleaved caspase-3-positive cells, and virus titers between different treatment groups were also statistically analyzed by using an unpaired Student *t* test. Differences in the body weights of untreated and 5Z-treated mice were analyzed using a repeated measures ANOVA test. Differences in the survival of untreated and 5Z-treated mice were statistically analyzed by using a Log-Rank test. The *p* value of < 0.05 was considered statistically significant. All statistics were performed with GraphPad Prism (GraphPad software 8.0.2) (https://www.graphpad.com/scientific-software/prism).

### Electronic supplementary material

Below is the link to the electronic supplementary material.

Supplementary Material 1

## Data Availability

No datasets were generated or analysed during the current study.
