# Supplementary material for: Influenza virus infection activates TAK1 to suppress RIPK3-independent apoptosis and RIPK1-dependent necroptosis
Source: Cell Commun Signal. 2024 Jul 23;22:372. doi: 10.1186/s12964-024-01727-2 (PMC11264382; doi:10.1186/s12964-024-01727-2)
Supplement: Supplementary file 1 — Supplementary Material 1 [file 12964_2024_1727_MOESM1_ESM.pdf]

Supplementary material

Supplementary Figures

Fig. S1

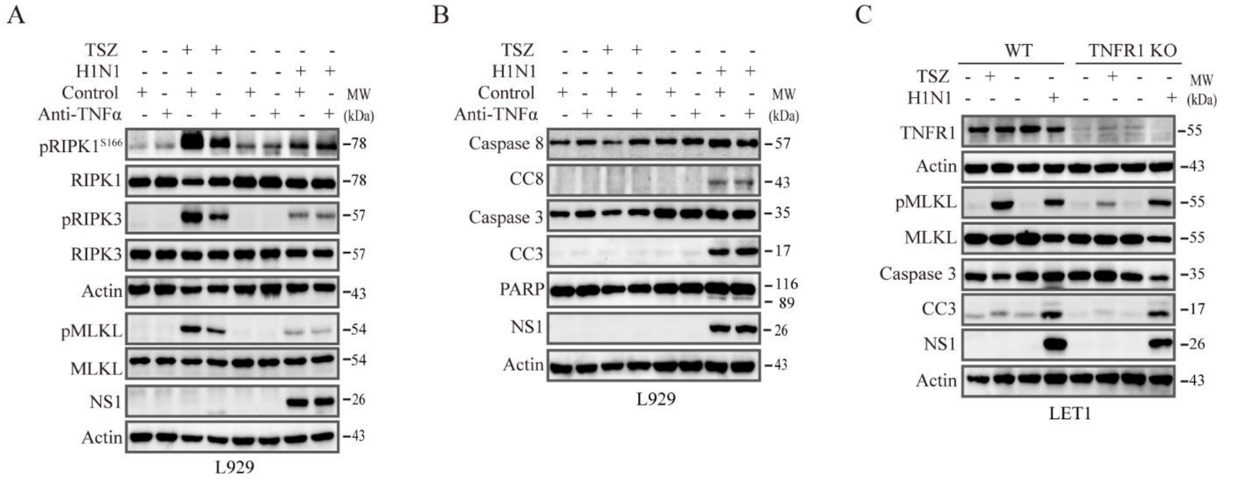

Fig. S1. H1N1 induces cell death independent of TNF-α. L929 cells were infected with H1N1 virus (5 MOI) and then incubated in the absence or presence of an anti-TNF-α neutralizing antibody or rat IgG as a negative control (2 μg each). Alternatively, L929 cells were stimulated with TSZ in which TNF-α (1 ng) was mixed with an anti-TNF-α antibody or rat IgG (2 μg each) and pre-incubated at 4°C for 1 hr. After incubation for 4 hr, cell lysates were prepared and analyzed for necroptosis-related protein phosphorylation (RIPK1^{S166}, RIPK3, and MLKL) and actin (**A**) and for apoptosis-related proteins (**B**) by Western blot. (**C**) Control and TNFR knockout LET1 cells were incubated in the absence or presence of TNF-α (20 ng/ml), Smac (100 nM), and Z-VAD (30 μM) for 12 hr or infected with H1N1 virus (2 MOI) for 18 hr. Cell lysates were analyzed for MLKL phosphorylation and caspase-3 cleavage, and the levels of TNFR, NS1, and actin by Western blot.

Fig. S2

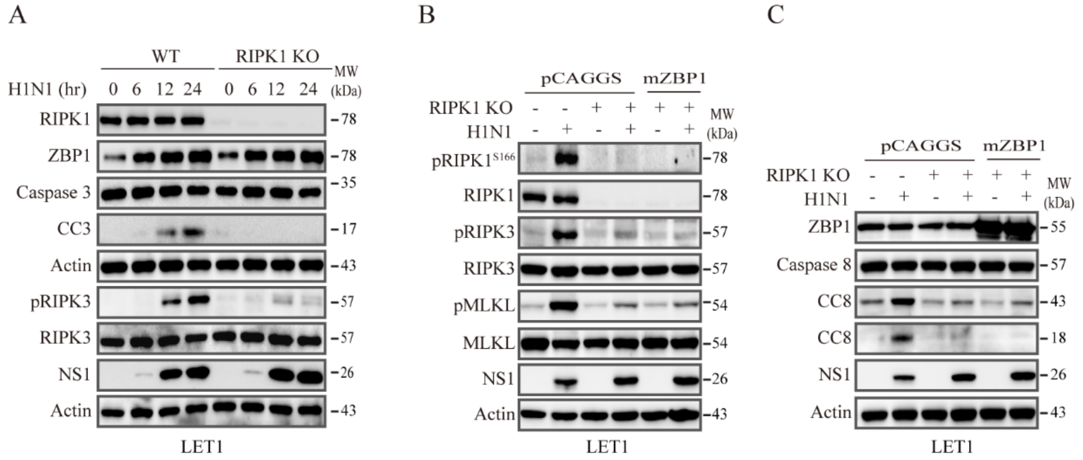

Fig. S2. Inhibition of IAV-induced cell death in RIPK1-deficient LET1 cells is unrelated to ZBP1. (**A**) Control and RIPK1 knockout LET1 cells were infected with 1 MOI of H1N1 virus and then incubated for 6, 12, 24 hr. Cell lysates were analyzed for the levels of ZBP1, RIPK3 phosphorylation, caspase-3 cleavage, NS1, and actin by Western blot. (**B & C**) Control and RIPK1 knockout LET1 cells were first transfected with the pCAGGS vector or the vector encoding the mZBP1 gene. After incubation for 24 hr, the cells were infected with 2 MOI of H1N1 virus and then incubated for another 24 hr. Cell lysates were analyzed for the levels of RIPK1, RIPK3, and MLKL phosphorylation and the levels of caspase-8 and caspase-3 cleavage, ZBP1, NS1, and actin by Western blot.

Fig. S3

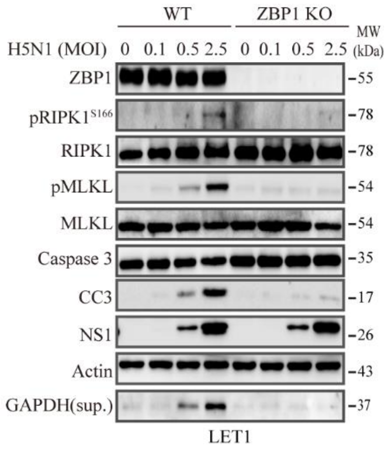

Fig. S3. ZBP1 deficiency abrogates H5N1 virus-induced cell death. Control and ZBP1 knockout LET1 cells were infected with the indicated MOI of H5N1 virus and incubated for 18 hr. Cell lysates were analyzed for the levels of RIPK1^{S166} and MLKL phosphorylation, caspase-3 cleavage, and GAPDH released into the conditioned media by Western blot.

Fig. S4

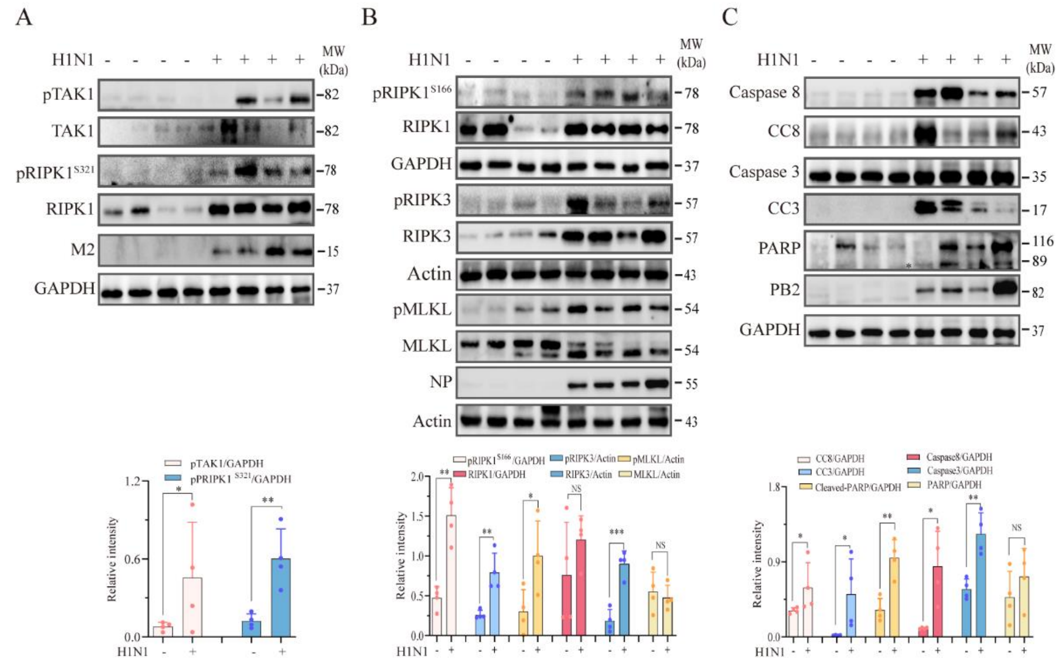

Fig. S4. IAV induces cell death in vivo. C57BL/6 mice were left uninfected or infected with H1N1 viruses (1000 pfu/mouse, 4 mice/group). Five days post-infection, mice were sacrificed. The lung tissue was collected and analyzed for the levels of TAK1 and RIPK1^{S321} phosphorylation (**A**), necroptosis-related protein phosphorylation (RIPK1^{S166}, RIPK3, and MLKL) and β-actin (**B**), and apoptosis-related proteins (**C**) by Western blot. The density of the phosphorylated protein or cleaved caspase bands was analyzed by using NIH Image-J software and normalized by the arbitrary units of their total protein bands or β-actin or GAPDH. Data are the mean ± SD from four mice per group, each lane represents one sample from one mouse. **p*<0.05, ***p*<0.01, ****p*<0.001, compared to the uninfected control.

sFig. 5

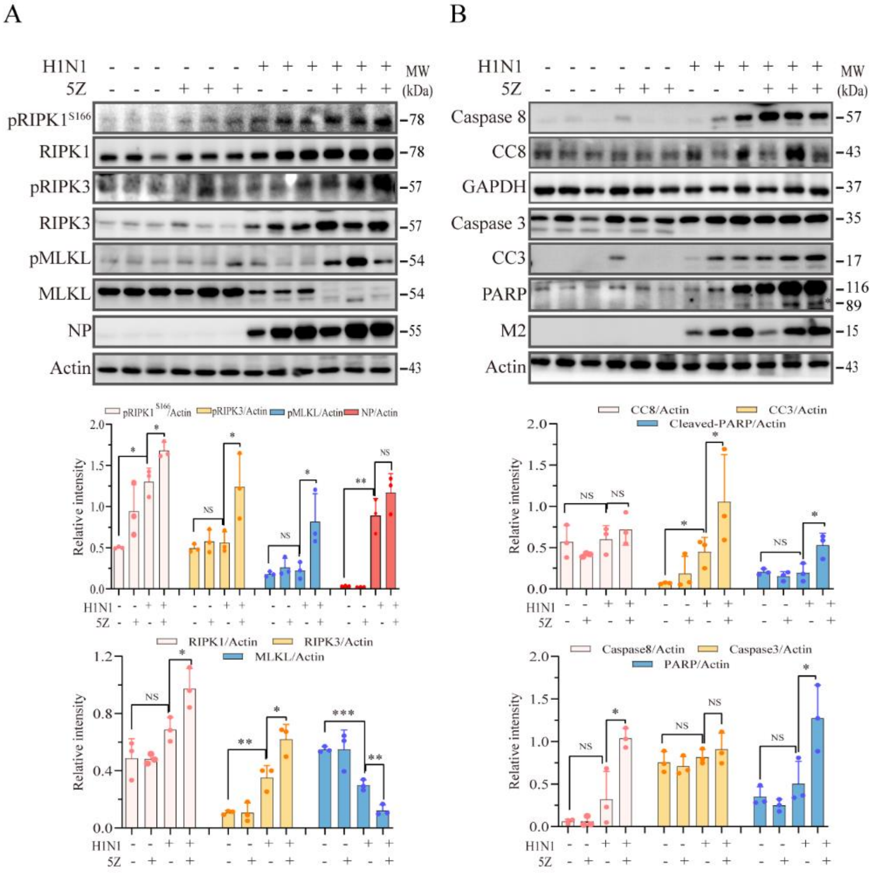

Fig. S5. 5Z enhances IAV-induced cell death in vivo. Female C57BL/6J mice (6-8-weeks old) were mock-infected or infected with with H1N1 viruses (1000 pfu/mouse, 3 mice/group). Twenty-four hours later, mice were treated with vehicle or 5Z (2 mg/kg body weight) daily for 2 days. Eight hours prior to sacrifice, mice were given a third dose of vehicle or 5Z. Mice were sacrificed at 80 hr post-infection (**A & B**). The lung tissue was collected and analyzed for the levels of necroptosis-related protein phosphorylation (RIPK1^{S166}, RIPK3, and MLKL) and actin (**A**), and apoptosis-related proteins (caspase-8, caspase-3, and PARP cleavage) (**B**) by Western blot. The density of the phosphorylated protein or cleaved caspase bands from 3 mice per group was analyzed using NIH Image-J software and normalized by the arbitrary units of β-actin. $^{*}p$<0.05, $^{**}p$<0.01.

Fig. S6

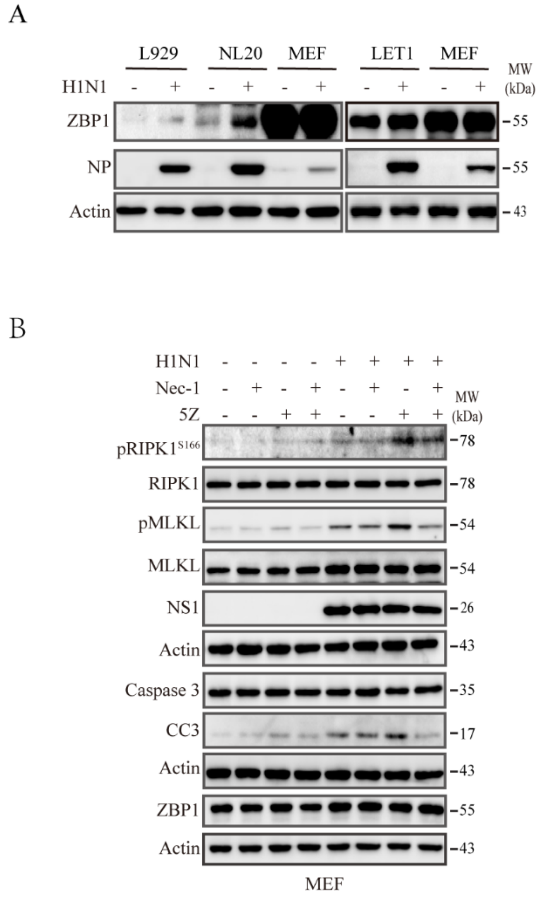

Fig. S6. MEF expresses ZBP1 at high levels. (A) L929, NL20, LET1 and MEF cells were left uninfected or infected with H1N1 virus (2 MOI). After incubation for 24 hr, cell lysates were prepared and analyzed for the levels of ZBP1, NP, and actin by Western blot. (**B**) Nec-1 does not inhibit H1N1 virus-induced cell death in MEF. MEF cells were infected with H1N1 virus (2 MOI). Nec-1 (50 μM) was added immediately after virus inoculation, whereas 5Z (0.5 μM) was added 12 hr post inoculation. Cells were then incubated for another 12 hr. Cell lysates were analyzed for the levels of MLKL and $RIPK1^{S166}$ phosphorylation, caspase-3 cleavage, ZBP1, and actin by Western blot.

Fig. S7

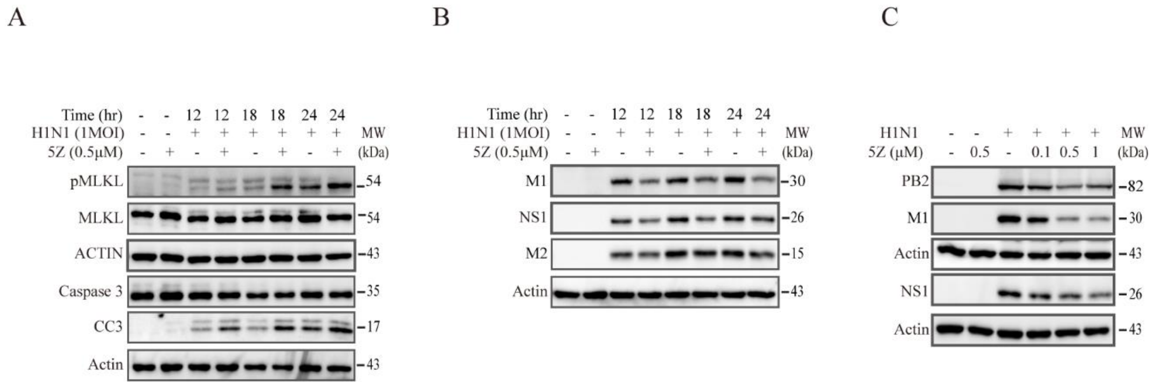

Fig. S7. 5Z inhibits IAV replication. L929 cells were infected with H1N1 virus (1 MOI). After incubation for 4 hr, 5Z (0.5 μM) was added. Cell lysates were prepared at 12, 18, or 24 hr post infection and analyzed for the levels of caspase 3 cleavage as well as MLKL phosphorylation (**A**) and the levels of viral M1, NS1, and M2 proteins (**B**) by Western blot. (**C**) L929 cells were infected with H1N1 viruses (1 MOI) and then incubated in the absence or presence of indicated concentration of 5Z. After incubation for 18 hr, cell lysates were prepared and analyzed for the levels of viral M1, NS1, and PB2 proteins by Western blot.

Supplementary Table

Table S1. Primers used for RT-qPCR

Gene	Forward primer	Reverse primer
IAV	5'-GCACTTGATATTGTGGATTCTTGATCG TCTT-3'	5'-GACAAAATGACCATCGTCAACATCCACA-3'
TNF-α	5'-CCCTCACACTCAGATCATCTTCT-3'	5'-GCTACGACGTGGGCTACAG-3'
IL-1β	5'-TGGACCTTCCAGGATGAGGACA-3'	5'-GTTCATCTCGGAGCCTGTAGTG-3'
CXCL10	5’-CCAAGTGCTGCCGTCATTTTC-3’	5’-GGCTCGCAGGGATGATTTCAA-3’
IL-6	5’-TGAGATCTACTCGGCAAACCTAGTG-3’	5’-CTTCGTAGAGAACAACATAAGTCAGATACC-3’
β-actin	5’-CATCCGTAAAGACCTCTATGCCAAC-3’	5’-ATGGAGCCACCGATCCACA-3’
